# Supplementary material for: Are weak or negative clinical recommendations associated with higher geographical variation in utilisation than strong or positive recommendations? Cross-sectional study of 24 healthcare services
Source: BMJ Open. 2021 May 10;11(5):e044090. doi: 10.1136/bmjopen-2020-044090 (PMC8112440; doi:10.1136/bmjopen-2020-044090)
Supplement: Supplementary data [file bmjopen-2020-044090supp001.pdf]

Additional file 1 Definitions and descriptions of the studied health care services and eligible populations

Category	Health care service	Service description and frequency	Eligible population	Recommendation for the health care service	Specific clinical explanatory variables	Clinical codes used for identification of the health care service
Screening	Colon cancer screening	Colonoscopy/ year	Anyone 50-69 years old	Colonoscopy should be done every 10 years for people 50-69 years old.	Previous treatment of cancer or inflammatory bowel disease, hospitalization with colon disease in the last year	Colonoscopy: 19.06 (TM Kapitel); G48% (DRG); 45.23, 45.25, 48.29.1%, 48.29.2% (CHOP)
	Breast cancer screening	Mammography/ year	50-74 years old women	Mammography should be done every 2 years for 50-74 years old women.	Previous treatment of breast or other cancer	Mamography: 39.1310, 39.1320, 39.1307, 39.1308, 39.1300, 39.1305, 39.1306 (TM); TZ
	Prostate cancer screening	Prostate-specific antigen (PSA) testing/ year	50-70 years old men	Early detection of prostate cancer (opportunistic screening) should be offered to the well-informed man.	Previous treatment of cancer, hospitalization with prostate disease in the last year	PSA testing: 1626.00 (Ana)
	Osteoporosis screening	Dual-energy x-ray absorptiometry (DXA)/ year	Women over 60 and with risk factors[a] of spontaneous fractures	DXA densitometry is recommended for postmenopausal women with spontaneous fractures or increased risk of them.	Presence of more than one risk factor	DXA densitometry: 39.1950, 39.2140, 39.2150, 39.2160 (TM)

Diagnosis	DM: HbA1c test	Glycated haemoglobin (HbA1c) test twice/ year	>18-year-old drug-treated diabetes patients	HbA1c test should be done for diabetes patients at least twice a year.	Oral diabetes medication or insulin	HbA1c test: 1363.00, 1363.01 (Ana)
	DM: renal function test	Albuminuria and serum creatinine tests/ year	>18-year-old drug-treated diabetes patients	Albuminuria and serum creatinine tests should be done for diabetes patients at least once a year.	Oral diabetes medication or insulin	Albuminuria: 1023.00, 1023.01, 1739.00, 1739.01, 1740.00, 1740.01 (Ana)<br>Serum creatinine: 1509.00, 1509.01 (Ana)
	DM: LDL test	Low-density lipoprotein (LDL) test/ year	19-75-year-old drug-treated diabetes patients	LDL test should be done for diabetes patients at least once a year.	Oral diabetes medication or insulin	LDL test: 1521.00 (Ana)<br>Total cholesterol test: 1230.00, 1230.01 (Ana)<br>HDL test: 1410.01, 1410.10 (Ana)<br>Triglycerides test: 1731.01, 1731.00 (Ana)
	DM: eye examination	Ophthalmologist visit/ year	>18-year-old drug-treated diabetes patients	Eye exam should be performed for diabetes patients at least once a year.	Oral diabetes medication or insulin	Outpatient visit with ophthalmologist: (sub group “Ophthalmologie” in Swiss care provider registry sasis.ch)
	TSH screening	Thyroid-stimulating hormone (TSH) test without T3 and T4 tests on the same day	>18-year-old persons without thyroid disease[b] and receiving TSH test	TSH should be measured as an initial screening test for hypo/hyperthyroidism, while T3 and T4 test should follow if TSH is abnormal.	-	TSH test: 1718.10 (Ana)<br>T3 or T4 test: 1732.00, 1720.00, 733.00, 1721.00 (Ana)

	POCR	Outpatient preoperative chest radiography (POCR) up to 2 months before surgery	>18-year-old patients with inpatient surgical procedures	Routine chest radiography is not recommended before surgery.	-	Chest radiography: 39.0190 (TM)
Primary prevention	Influenza vaccination	Influenza outpatient vaccination/ year	People over 65 years old or with a specified chronic condition[c]	People over 65 years old and patients with chronic conditions, specified by Federal Office of Public Health, should be vaccinated against influenza every year.	Hospitalization with pneumonia in the last year	Influenza vaccination: J07BB02 (ATC)
Treatment	Benzodiazepines	Cumulative prescription of benzodiazepines (BZD) for >8 weeks/ year	Anyone over 65 years old	Long-term use of benzodiazepines and other hypnotics is discouraged for old patients.	Treated epilepsy, stay in a nursing home in the last year, hospitalization in the last year with a diagnosis indicative of justified benzodiazepine use	Benzodiazepines and other hypnotics: N03AE01, N05BA%, N05CD%, N05BB%, N05BE%, N05CA%, N05CB%, N05CC%, N05CF%, N05CH%, N05CM%, N05CX% (ATC)
	Proton pump inhibitors	Cumulative prescription of proton pump inhibitors (PPI) or H2 histamine receptor antagonists (H2) for >8 weeks/ year	>18-year-old persons receiving PPI or H2 drugs	PPI should not be used at maximal dose for prolonged periods of time.	-	PPI or H2: A02BC%, A02BD%, M01AE52, A02BA% (ATC)
	Inpatient procedures	Specified surgical procedures[d] done in	>18-year-old patients with	If none of the special conditions apply, certain surgical	-	

		the outpatient setting	specified surgical procedures (either as in- or outpatient)	procedures should be done in the outpatient setting.		
	Caesarean section	Caesarean section (C-section)	>18-year-old women giving birth without absolute indications[e] for C-section	C-section should not be performed unless absolute or relative indications are present.	-	C-section: 74.0%, 74.1%, 74.2%, 74.4%, 74.99 (CHOP); O01A, O01B, O01C, O01D, O01E, O01F (DRG); 22.2120, 22.2130, 22.2410, 22.2420 (TM)
Secondary prevention	AMI: aspirin	Aspirin prescription within 2 weeks after acute myocardial infarction (AMI)	>18-year-old patients with AMI[f]	All myocardial infarction patients should take aspirin long-term.	Hospitalization for stroke or bleeding event or prescribed anticoagulation in the last year	Aspirin: B01AC06 (ATC)
	AMI: statin	High-dose statin prescription within 2 weeks after AMI	>18-year-old patients with AMI [f]	All myocardial infarction patients should get statins long-term.	Hospitalization for stroke in the last year	High-dose statins: C10AA05, C10AA07 (ATC)
	AMI: beta-blocker	Beta-blocker prescription within 2 weeks after AMI	>18-year-old patients with AMI [f]	All myocardial infarction patients with heart failure or impaired function should get beta-blockers long-term.	Hospitalization with heart failure diagnosis in the last year	Beta-blockers: C07% (ATC)
	AMI: ACE/ARB	Angiotensin-converting enzyme (ACE) or angiotensin	>18-year-old patients with AMI [f]	All myocardial infarction patients with heart failure or impaired function should get	-	ACE or ARB medication: C09% (ATC)

		receptor blocker (ARB) antihypertensive medication prescription within 2 weeks after AMI		ACE or ARB antihypertensive medication long-term.		
	AMI: P2Y12 inhibitors	P2Y12 antiplatelet drug[g] prescription within 2 weeks after AMI	>18-year-old patients with AMI [f]	All myocardial infarction patients should get P2Y12 antiplatelet drugs for at least 1-12 months according to the bleeding risk profile and AMI treatment.	Hospitalization for a bleeding event or prescribed anticoagulation in the last year	P2Y12 drugs: B01AC04, B01AC22, B01AC24 (ATC)
	PPI with NSAID	PPI prescription within 1 month or up to 3 months before initial long-term nonsteroidal anti-inflammatory drug (NSAID) prescription	>18-year-old patients with a cumulative NSAID prescription of >8 weeks at maximal dose	Patients taking long-term NSAID and with risk factors for gastric ulcer[h] should also take PPI.	Concurrent use of antiplatelet, anticoagulation drugs or oral glucocorticoids, hospitalization for bleeding event in the last year.	NSAID: M01A% (ATC)<br>PPI: A02BC%, A02BD%, M01AE52 (ATC)
	PAD: statin	Prescription of statins within 3 months after peripheral artery disease (PAD) identification	>18-year-old patients undergoing diagnostic or treatment	Statins are recommended for all patients with PAD.	-	Statins: C10AA%, C10B% (ATC)

			procedures for PAD[i]			
	Afib: anticoagulation	Oral anticoagulation prescription within 2 weeks after atrial fibrillation (Afib) identification	>18-year-old patients with atrial fibrillation diagnosis and additional risk factors[j]	All patients with atrial fibrillation should be prescribed oral anticoagulation for embolic events prevention according to the CHA_2DS_2-VASc score.	-	Oral anticoagulation: B01AE07, B01AF01, B01AF02, B01AF03, B01AA04, B01AA07 (ATC)
	GCC with new DMARD	Glucocorticoid (GCC) prescription within 1 month or up to 3 months before disease-modifying antirheumatic drug (DMARD) prescription	>18-year-old patients with a new prescription of DMARD by a rheumatologist	Short-term glucocorticoids should be taken with newly prescribed DMARD.	-	Glucocorticoids: H02% (ATC) DMARD: L01BA01, L04AX03, M01CX01, L04AA13, M01CX02, P1BA02, P01BA01, M01CC01, L01AA01, M01CB01, L04AX01 (ATC)

a. Recent distal radius, proximal humerus, vertebral or femoral fracture, use of drugs increasing the risk of osteoporosis, use of oral glucocorticoids, diabetes, ankylosing spondylitis, osteogenesis imperfecta, rheumatoid arthritis, inflammatory bowel disease, Cushing's disease, alcohol or nicotine abuse, chronic liver disease, gastrectomy, malnutrition, hypogonadism, hyper- or hypothyroidism, and hyperparathyroidism. Patients currently treated or diagnosed with osteoporosis were excluded.

b. Hyperthyroidism, hypothyroidism, goitre or thyroiditis.

c. Cardiovascular disease, chronic pulmonary disease, diabetes, chronic liver disease, renal failure, immune deficiency, systemic neurologic disorders.

d. Varicose veins ligation and stripping, surgical procedures of haemorrhoids, inguinal hernia and cervix, knee arthroscopy and meniscectomy, tonsillectomy.

e. Placental, umbilical cord or fetal pathology, HIV or genital HSV infection, or multiple pregnancy.

f. Inpatient treatment with a diagnosis of acute myocardial infarction (AMI).

g. Clopidogrel, prasugrel or ticagrelor.

Ulyte A, *et al. BMJ Open* 2021; 11:e044090. doi: 10.1136/bmjopen-2020-044090

BMJ Publishing Group Limited (BMJ) disclaims all liability and responsibility arising from any reliance placed on this supplemental material which has been supplied by the author(s)

h. Concurrent use of antiplatelet, anticoagulant drugs, oral glucocorticoids or recent hospitalization with any major bleeding.

i. Peripheral artery disease (PAD) or carotid stenosis diagnosed during an inpatient stay, amputation of lower or upper extremity, thrombectomy, stenting or other procedures in peripheral arteries, specialized diagnostic ultrasound, magnetic resonance tomography (MRI) angiography, computer tomography (CT) angiography or angiography of peripheral arteries.

j. Risk factors (congestive heart failure, hypertension, age 65-74 or ≥75 years old, diabetes, previous stroke, transient ischemic attack, or thromboembolism, cardiovascular disease, female sex) were extracted from available claims data and summed according to CHA2DS2-VASc score. Patients with CHA2DS2-VASc score of ≥2 for males and ≥3 for females were included.

DM – diabetes mellitus, HbA1c – Glycated haemoglobin, LDL – low density lipid, TSH – thyroid-stimulating hormone, T3 and T4 – triiodothyronine and thyroxine, POCR – preoperative chest radiography, BZD – benzodiazepines, PPI – proton pump inhibitors, H2 – H2 histamine receptor antagonists, C-section – Caesarean section, AMI – acute myocardial infarction, ACE/ARB – angiotensin-converting enzyme inhibitors or angiotensin II receptor blockers, NSAID – nonsteroidal anti-inflammatory drugs, PAD – peripheral artery disease, Afib – atrial fibrillation, GCC – glucocorticosteroid drugs, DMARD – disease-modifying antirheumatic drug.

Ana – Analysenliste, Swiss outpatient laboratory test codes; ATC - Anatomical Therapeutic Chemical Classification System, code and quantity of a prescription drug; CHOP - Schweizerische Operationsklassifikation, a classification of inpatient procedures; DRG - Swiss Diagnosis Related Groups, a classification of inpatient cases, based on diagnoses, procedures and other clinical information; ICD - International Classification of Diseases, 10th revision, German Modification, codes for primary and secondary diagnoses for each hospitalization episode of an inpatient; TM – Tarmed, Swiss classification of outpatient procedures and services; TM Kapitel – Tarmed chapter codes; TZ – Tarifziffer, further codes representing reimbursement of screening services within cantonal breast cancer screening programs.

Ulyte A, *et al*. *BMJ Open* 2021; 11:e044090. doi: 10.1136/bmjopen-2020-044090
