# Supplementary material for: Are weak or negative clinical recommendations associated with higher geographical variation in utilisation than strong or positive recommendations? Cross-sectional study of 24 healthcare services
Source: BMJ Open. 2021 May 10;11(5):e044090. doi: 10.1136/bmjopen-2020-044090 (PMC8112440; doi:10.1136/bmjopen-2020-044090)
Supplement: Supplementary data [file bmjopen-2020-044090supp002.pdf]

Additional file 2 Algorithm and criteria for the assessment of the strength of recommendation

N of authors	Steps	
Single	1.	Identify the relevant medical societies for each selected health care service.
	2.	Look up the European or international medical societies' websites and journals, and identify relevant guidelines published before 2014. In addition, look up if Swiss federal legislation guidelines exist by 2014.
	3.	If none found, look up American medical society and identify relevant guidelines published before 2014.
	4.	If none found, consider the recommendation **weak**.
In duplicate	5.	Once the guideline and the recommendation statement are located, classify the recommendation into strong or weak[a].
	-	**Strong recommendation** implies that the desirable effects of adherence to a recommendation outweigh the undesirable effects.
	-	That means that most informed patients would choose the recommended management and that clinicians can structure their interactions with patients accordingly.
	-	For clinicians, that would mean that most patients should receive the recommended course of action.<br>For patients, that would mean that most people in such a situation would want the recommended course of action and only a small proportion would not; patients should request discussion if the intervention is not offered.
	-	**Weak recommendation** implies that the desirable effects of adherence to a recommendation probably outweigh the undesirable effects, but the guideline panel is less confident.
	-	Thus, a weak recommendation is conditional or optional, and means that patients' choices will vary according to their values and preferences, and clinicians must ensure that patients' care is in keeping with their values and preferences.
	-	For clinicians, that would mean that they should recognize that different choices will be appropriate for different patients and that they must help each patient to arrive at a management decision consistent with her or his values and preferences.<br>For patients, that would mean that most people in such situation would want the recommended course of action, but many would not.

a adapted from: Guyatt GH, Oxman AD, Kunz R, Falck-Ytter Y, Vist GE, Liberati A, et al. GRADE: Going from Evidence to Recommendations. BMJ 2008;336:1048–51.

Ulyte A, *et al. BMJ Open* 2021; 11:e044090. doi: 10.1136/bmjopen-2020-044090
