# Supplementary material for: Are weak or negative clinical recommendations associated with higher geographical variation in utilisation than strong or positive recommendations? Cross-sectional study of 24 healthcare services
Source: BMJ Open. 2021 May 10;11(5):e044090. doi: 10.1136/bmjopen-2020-044090 (PMC8112440; doi:10.1136/bmjopen-2020-044090)
Supplement: Supplementary data [file bmjopen-2020-044090supp003.pdf]

Additional file 3 Algorithm and criteria for the assessment of the direction of recommendation

N of authors	Steps	
In duplicate	1.	Once the guideline and the recommendation statement are located (see Additional file 2), classify the recommendation into positive and negative.
	-	**Positive** recommendation encourages the use of a health care service in a given population.
	-	**Negative** recommendation discourages the use of a health care service in a given population (e.g., contains negative indicatory words, such as *not, no, never*)

Ulyte A, *et al. BMJ Open* 2021; 11:e044090. doi: 10.1136/bmjopen-2020-044090
