# Supplementary material for: Are weak or negative clinical recommendations associated with higher geographical variation in utilisation than strong or positive recommendations? Cross-sectional study of 24 healthcare services
Source: BMJ Open. 2021 May 10;11(5):e044090. doi: 10.1136/bmjopen-2020-044090 (PMC8112440; doi:10.1136/bmjopen-2020-044090)
Supplement: Supplementary data [file bmjopen-2020-044090supp004.pdf]

Additional file 4 List of guidelines selected for the study, describing the services analysed

Recommendation	Reference	Comment
Colon cancer screening	[1]	
Breast cancer screening	[2]	
Prostate cancer screening	[3]	
Osteoporosis screening	[4]	
DM: HbA1c test	[5]	
DM: renal function test	[5]	
DM: LDL test	[5]	
DM: eye examination	[5]	
TSH screening	[6,7]	
POCR	[8]	
Influenza vaccination	[9]	
Benzodiazepines	[10]	
Proton pump inhibitors	-	Swiss national guideline since 2016 [11]
Inpatient procedures	-	Swiss federal regulation exists from 2019 [12]
Caesarean section	-	Swiss national guideline since 2015 [13]
AMI: aspirin	[14,15]	
AMI: statin	[14,15]	
AMI: beta-blocker	[14,15]	
AMI: ACE/ARB	[14,15]	
AMI: P2Y12 inhibitors	[14,15]	
PPI with NSAID	[16]	
PAD: statin	[17]	
Afib: anticoagulation	[18]	
GCC with new DMARD	[19]	

DM – diabetes mellitus, HbA1c – Glycated haemoglobin, LDL – low density lipid, TSH – thyroid-stimulating hormone, POCR – preoperative chest radiography, AMI – acute myocardial infarction, ACE/ARB – angiotensin-converting enzyme inhibitors or angiotensin II receptor blockers, PPI – proton pump inhibitors, NSAID – nonsteroidal anti-inflammatory drugs, PAD – peripheral artery disease, Afib – atrial fibrillation, GCC – glucocorticosteroid drugs, DMARD – disease-modifying antirheumatic drug.

1. Lansdorp-Vogelaar I, Karsa L. European guidelines for quality assurance in colorectal cancer screening and diagnosis. First Edition – Introduction. Endoscopy. 2012 Sep 25;44(S 03):SE15–30.
2. Screening for Breast Cancer: U.S. Preventive Services Task Force Recommendation Statement. Ann Intern Med. 2009 Nov 17;151(10):716.
3. Heidenreich A, Bellmunt J, Bolla M, Joniau S, Mason M, Matveev V, et al. EAU Guidelines on Prostate Cancer. Part 1: Screening, Diagnosis, and Treatment of Clinically Localised Disease. Eur Urol. 2011 Jan;59(1):61–71.

Ulyte A, *et al. BMJ Open* 2021; 11:e044090. doi: 10.1136/bmjopen-2020-044090

4. Kanis JA, McCloskey E V., Johansson H, Cooper C, Rizzoli R, Reginster J-Y. European guidance for the diagnosis and management of osteoporosis in postmenopausal women. Osteoporos Int. 2013 Jan 19;24(1):23–57.
5. Rydén L, Grant PJ, Anker SD, Berne C, Cosentino F, Danchin N, et al. ESC Guidelines on diabetes, pre-diabetes, and cardiovascular diseases developed in collaboration with the EASD. Eur Heart J. 2013 Oct 14;34(39):3035–87.
6. Bahn RS, Burch HB, Cooper DS, Garber JR, Greenlee MC, Klein I, et al. Hyperthyroidism and Other Causes of Thyrotoxicosis: Management Guidelines of the American Thyroid Association and American Association of Clinical Endocrinologists. Thyroid. 2011 Jun;21(6):593–646.
7. Garber J, Cobin R, Gharib H, Hennessey J, Klein I, Mechanick J, et al. Clinical Practice Guidelines for Hypothyroidism in Adults: Cosponsored by the American Association of Clinical Endocrinologists and the American Thyroid Association. Endocr Pract. 2012 Nov;18(6):988–1028.
8. Choosing Wisely. American College of Surgeons. Admission pre-op chest x-rays. September 4, 2013. Available from: https://www.choosingwisely.org/clinician-lists/american-college-surgeons-admission-or-preop-chest-x-ray-on-ambulatory-patients/ Accessed on May 11, 2020
9. Swiss Federal Office of Public Health. Recommendations for Influenza Vaccination (Empfehlungen zur Grippeimpfung). 2011.
10. Choosing Wisely. American Geriatrics Society. Benzodiazepines sedative hypnotics for insomnia in older adults. February 21, 2013. Available from: https://www.choosingwisely.org/clinician-lists/american-geriatrics-society-benzodiazepines-sedative-hypnotics-for-insomnia-in-older-adults/ Accessed on May 11, 2020
11. Swiss Society of General Internal Medicine. Smarter Medicine. Top-5 List for Ambulatory Care. 2016. Available from: https://www.smartermedicine.ch/de/top-5-listen/ambulante-allgemeine-innere-medizin.html Accessed on May 11, 2020
12. Swiss Federal Office of Public Health. “Outpatient instead of Inpatient” [Änderung der Krankenpflege-Leistungsverordnung (KLV) betreffend «Ambulant vor Stationär»]. Available from: https://www.bag.admin.ch/bag/de/home/versicherungen/krankenversicherung/krankenversicherung-revisionsprojekte/konsultation-ambulant-vor-stationaer.html Accessed on May 11, 2020
13. Hoesli I, Alma-Stucki S El, Drack G, Girard T, Irion O, Schulzke S, et al. Guideline Sectio Caesarea. 2015;1–20. Available from: https://www.sggg.ch/fachthemen/guidelines/ Accessed on May 11, 2020
14. Hamm CW, Bassand J-P, Agewall S, Bax J, Boersma E, Bueno H, et al. ESC Guidelines for the management of acute coronary syndromes in patients presenting without persistent ST-segment elevation: The Task Force for the management of acute coronary syndromes (ACS) in patients presenting without persistent ST-segment elevatio. Eur Heart J. 2011 Dec 1;32(23):2999–3054.
15. Steg PG, James SK, Atar D, Badano LP, Lundqvist CB, Borger MA, et al. ESC Guidelines for the management of acute myocardial infarction in patients presenting with ST-segment elevation. Eur Heart J. 2012 Oct 1;33(20):2569–619.
16. Recommendations for use of selective and nonselective nonsteroidal antiinflammatory drugs: An American College of Rheumatology white paper. Arthritis Rheum. 2008 Aug 15;59(8):1058–73.

17. Tendera M, Aboyans V, Bartelink M-L, Baumgartner I, Clement D, Collet J-P, et al. ESC Guidelines on the diagnosis and treatment of peripheral artery diseases: Document covering atherosclerotic disease of extracranial carotid and vertebral, mesenteric, renal, upper and lower extremity arteries * The Task Force on the Diagnosis and Treat. Eur Heart J. 2011 Nov 2;32(22):2851–906.
18. Camm AJ, Lip GYH, De Caterina R, Savelieva I, Atar D, Hohnloser SH, et al. 2012 focused update of the ESC Guidelines for the management of atrial fibrillation. Eur Heart J. 2012 Nov 1;33(21):2719–47.
19. Smolen JS, Landewé R, Breedveld FC, Buch M, Burmester G, Dougados M, et al. EULAR recommendations for the management of rheumatoid arthritis with synthetic and biological disease-modifying antirheumatic drugs: 2013 update. Ann Rheum Dis. 2014 Mar;73(3):492–509.
