# Supplementary material for: Are weak or negative clinical recommendations associated with higher geographical variation in utilisation than strong or positive recommendations? Cross-sectional study of 24 healthcare services
Source: BMJ Open. 2021 May 10;11(5):e044090. doi: 10.1136/bmjopen-2020-044090 (PMC8112440; doi:10.1136/bmjopen-2020-044090)
Supplement: Supplementary data [file bmjopen-2020-044090supp005.pdf]

Additional file 5 Geographic variation of the health care services grouped by strength and direction of recommendations, and service category

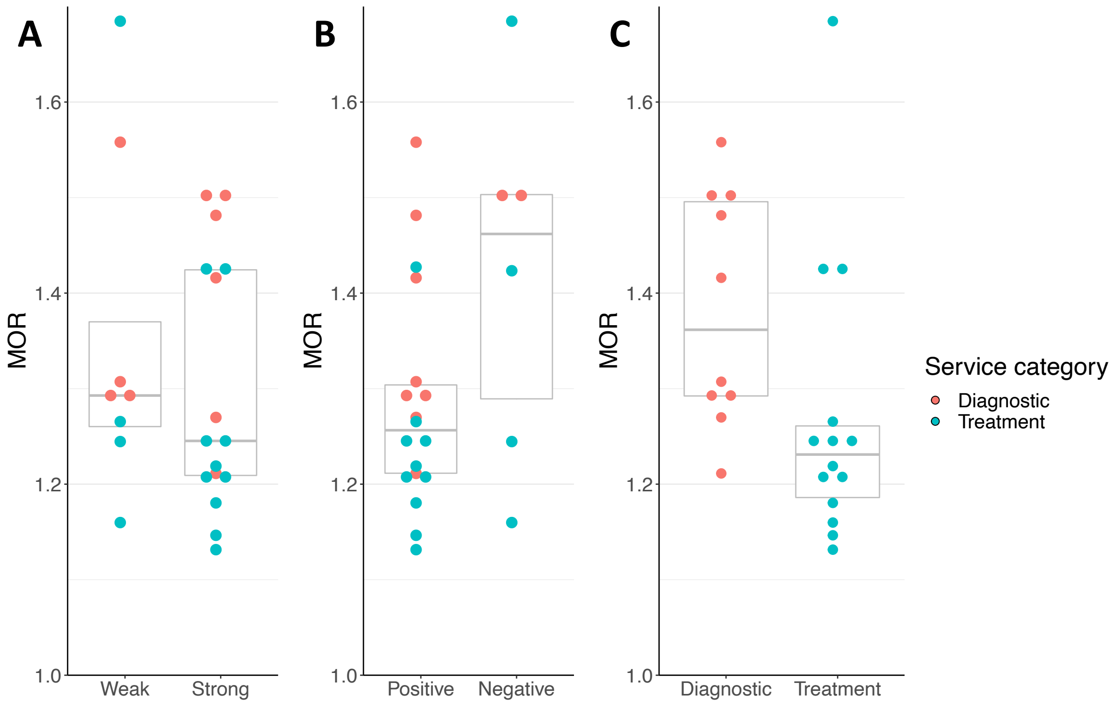

A Weak and strong recommendations; B Positive and negative recommendations; C Diagnostic and treatment services. MOR – median odds ratio. Boxplots depict the interquartile range of values (upper and lower hinges), and the median value.

Based on Welch's t-test, the difference in mean variances [95CI%] of diagnostic and treatment services was 0.04 [-0.01, 0.11], and the difference in mean MOR was 0.11 [-0.01, 0.23].

Ulyte A, *et al. BMJ Open* 2021; 11:e044090. doi: 10.1136/bmjopen-2020-044090
