# Supplementary material for: Are weak or negative clinical recommendations associated with higher geographical variation in utilisation than strong or positive recommendations? Cross-sectional study of 24 healthcare services
Source: BMJ Open. 2021 May 10;11(5):e044090. doi: 10.1136/bmjopen-2020-044090 (PMC8112440; doi:10.1136/bmjopen-2020-044090)
Supplement: Author's manuscript [file bmjopen-2020-044090.draft_revisions.pdf]

BMJ Open is committed to open peer review. As part of this commitment we make the peer review history of every article we publish publicly available.

When an article is published we post the peer reviewers' comments and the authors' responses online. We also post the versions of the paper that were used during peer review. These are the versions that the peer review comments apply to.

The versions of the paper that follow are the versions that were submitted during the peer review process. They are not the versions of record or the final published versions. They should not be cited or distributed as the published version of this manuscript.

BMJ Open is an open access journal and the full, final, typeset and author-corrected version of record of the manuscript is available on our site with no access controls, subscription charges or pay-per-view fees (http://bmjopen.bmj.com).

If you have any questions on BMJ Open's open peer review process please email info.bmjopen@bmj.com

BMJ Open

Are weak or negative clinical recommendations associated with higher geographic variation in utilization than strong or positive recommendations? Cross-sectional study of 24 health care services

Journal:	*BMJ Open*
Manuscript ID	bmjopen-2020-044090
Article Type:	Original research
Date Submitted by the Author:	24-Aug-2020
Complete List of Authors:	Ulyte, Agne; University of Zurich, EBPI<br>Wei, Wenjia; University of Zurich, EBPI<br>Gruebner, Oliver; University of Zurich, EBPI<br>Bähler, Caroline ; Helsana Group, Department of Health Sciences<br>Brüngger, Beat; Helsana Group, Department of Health Sciences<br>Blozik, Eva; Helsana Group, Department of Health Sciences<br>von Wyl, Viktor ; University of Zurich, EBPI<br>Schwenkglenks, M; University of Zurich<br>Dressel, Holger; University of Zurich
Keywords:	EPIDEMIOLOGY, Organisation of health services < HEALTH SERVICES ADMINISTRATION & MANAGEMENT, Protocols & guidelines < HEALTH SERVICES ADMINISTRATION & MANAGEMENT, HEALTH SERVICES ADMINISTRATION & MANAGEMENT

SCHOLARONE™
Manuscripts

I, the Submitting Author has the right to grant and does grant on behalf of all authors of the Work (as defined in the below author licence), an exclusive licence and/or a non-exclusive licence for contributions from authors who are: i) UK Crown employees; ii) where BMJ has agreed a CC-BY licence shall apply, and/or iii) in accordance with the terms applicable for US Federal Government officers or employees acting as part of their official duties; on a worldwide, perpetual, irrevocable, royalty-free basis to BMJ Publishing Group Ltd ("BMJ") its licensees and where the relevant Journal is co-owned by BMJ to the co-owners of the Journal, to publish the Work in this journal and any other BMJ products and to exploit all rights, as set out in our licence.

The Submitting Author accepts and understands that any supply made under these terms is made by BMJ to the Submitting Author unless you are acting as an employee on behalf of your employer or a postgraduate student of an affiliated institution which is paying any applicable article publishing charge ("APC") for Open Access articles. Where the Submitting Author wishes to make the Work available on an Open Access basis (and intends to pay the relevant APC), the terms of reuse of such Open Access shall be governed by a Creative Commons licence – details of these licences and which Creative Commons licence will apply to this Work are set out in our licence referred to above.

Other than as permitted in any relevant BMJ Author's Self Archiving Policies, I confirm this Work has not been accepted for publication elsewhere, is not being considered for publication elsewhere and does not duplicate material already published. I confirm all authors consent to publication of this Work and authorise the granting of this licence.

For peer review only

Are weak or negative clinical recommendations associated with higher geographic variation in utilization than strong or positive recommendations? Cross-sectional study of 24 health care services

Authors

Agne Ulyte[a]*, Wenjia Wei[a], Oliver Gruebner[a,b], Caroline Bähler[a,c], Beat Brüngger[a,c], Eva Blozik[c,d], Viktor von Wyl[a], Matthias Schwenkglenks[a], Holger Dressel[e]

Affiliations

a - Department of Epidemiology, Epidemiology, Biostatistics & Prevention Institute, University of Zurich, Hirschengraben 84, 8001 Zurich, Switzerland

b – Department of Geography, University of Zurich, Winterthurerstrasse 190, 8057 Zurich, Switzerland

Hospital Zurich, Hirschengraben 84, 8001 Zurich, Switzerland

c - Department of Health Sciences, Helsana Group, Zürichstrasse 130, 8600 Dübendorf, Switzerland

d - Institute of Primary Care, University of Zurich and University Hospital Zurich, Pestalozzistrasse 24, 8091 Zürich, Switzerland

e – Division of Occupational and Environmental Medicine, Department of Epidemiology, Epidemiology, Biostatistics & Prevention Institute, University of Zurich and University Hospital Zurich, Hirschengraben 84, 8001 Zurich, Switzerland

*corresponding author

Agne Ulyte

Email: agne.ulyte@uzh.ch

Phone: +41 44 63 44681

For peer review only - http://bmjopen.bmj.com/site/about/guidelines.xhtml

25 **Address:** University of Zurich, Epidemiology, Biostatistics and Prevention Institute (EBPI), Hirschengraben 84,

26 8001 Zurich, Switzerland

For peer review only

Abstract

Objectives

When research evidence is lacking, patient and provider preferences, expected to vary geographically, might have a stronger role in clinical decisions. In this study, we investigated whether the strength or the direction of recommendation is associated with the degree of geographic variation in utilization.

Design

In this cross-sectional study, we selected 24 services following a comprehensive approach. The strength and direction of recommendations were assessed in duplicate. Multilevel models were used to adjust for demographic and clinical characteristics and estimate unwarranted variation.

Setting

Observational study of claims to mandatory health insurance in Switzerland in 2014.

Participants

Enrolees eligible for the 24 health care services.

Primary outcome measures

The resulting variances of regional random effects, also expressed as median odds ratio (MOR). Services grouped by strength and direction of recommendations were compared with Welch's t-test.

Results

The sizes of the eligible populations ranged from 1992 to 409 960 patients. MOR ranged between 1.13 for aspirin in secondary prevention of myocardial infarction to 1.68 for minor surgical procedures performed in inpatient instead of outpatient settings. Services with weak recommendations had a negligibly higher variance and MOR (difference in means [95CI%] 0.03 [-0.06, 0.11] and 0.05 [-0.11, 0.21]). Services with negative recommendations had a slightly higher variance and MOR (difference in means [95CI%] 0.07 [-0.03, 0.18] and 0.14 [-0.06, 0.34]).

Conclusions

In this exploratory study, the geographic variation in the utilization of services associated with strong versus weak recommendations was not substantially different. The geographic variation of services associated with negative recommendations was slightly higher than for those with positive recommendations. The relationships

For peer review only - http://bmjopen.bmj.com/site/about/guidelines.xhtml

between the strength and direction of recommendations and the variation may be indirect or modified by other characteristics of services. As initiatives discouraging low-value care are gaining attention worldwide, these findings may inform future research in this area.

Strengths and limitations of this study

- Although the strength and direction of recommendations is generally expected to influence the variation in clinical decisions, this is the first study to analyse this relationship quantitatively.
- The effect of the strength and direction of a recommendation on the geographic variation in health care utilization was assessed within a comprehensive set of 24 health care services.
- Unwarranted variation of the services utilization was extracted with a single standard approach.
- Indirect relationship and modifiers of the effect could not be studied.

Keywords: geographic variation in health care; unwarranted variation; clinical recommendations; clinical practice guidelines; evidence-based medicine; low-value care.

For peer review only

Background

According to the evidence-based medicine (EBM) framework, clinical decisions should be guided by research evidence, clinical circumstances, and patient preferences, and be integrated with clinical expertise [1]. If evidence is weak or lacking, patient preferences and clinical expertise have a particularly strong role in the decision [2, 3]. In a clinical practice guideline, such a situation would be reflected by a weak recommendation [2]. As patient preferences tend to vary geographically [4], and physician practice styles are also significantly influenced by the region of practice [5, 6], clinical decisions associated with less conclusive research evidence or weak recommendations may have higher geographic variation.

Surprisingly, there is little direct evidence whether weak recommendations are, in fact, associated with higher variation. The few available studies focused on a single specialty and did not quantify the variation in a uniform way, complicating the comparison of results [7, 8]. Therefore, despite many studies highlighting the substantial geographic variation in the utilization of various health care services [9–11], it is not clear, what role the components of the EBM framework play for this variation.

A second potential contributor to different services having different degrees of geographic variation is the direction of recommendation: positive (prescriptive) or negative (proscriptive), as in Choosing Wisely recommendations [12]. Negative recommendations usually concern long-used low-value practices and are based on the lack of supporting evidence or evidence of harms [12–14]. In contrast to positive recommendations, which often introduce new services or indications, negative recommendations usually challenge existing practices that are justified primarily by clinical expertise and judgement, expected to vary regionally [4]. Positive and negative recommendations have different perceived barriers to their implementation [15], which could contribute to different variation patterns as well. However, no study has directly compared the geographic variation associated with positive and negative recommendations.

The primary aim of this study was to assess whether health care services with weak recommendations are associated with higher geographic variation in utilization. In addition, a secondary aim was to test the association of geographic variation with the direction of the recommendation.

For peer review only

Methods

Study hypotheses

Although this is primarily an explorative study, we formulated two specific hypotheses. The primary hypothesis of the study was that health care services with weaker evidence, as reflected in weak recommendations in clinical guidelines, would have higher geographic variation in utilization than those with strong recommendations. The secondary hypothesis was that services with negative (proscriptive) recommendations would have higher geographic variation compared to those with positive (prescriptive) recommendations.

Selection of studied health care services

This study was part of a project assessing the geographic variation of the utilization of a set of health care services in Switzerland [16]. Studied health care services were translated from selected recommendation statements in clinical practice guidelines, following a systematic approach. We collected clinical practice guidelines of Swiss, European, and applicable international medical societies, used in Switzerland and guiding the care for major non-communicable diseases (as defined by the Swiss Federal Office of Public Health, FOPH [17]). Recommendation statements from selected clinical practice guidelines were considered pragmatically by the authors according to their clinical relevance, the expected frequency of service use, and the size of the eligible population. Identified recommended or discouraged services were then screened for feasibility of measuring the utilization in eligible populations with Swiss health insurance claims data, based on an approach described earlier [18].

We aimed for the selected services to reflect both strong and weak, positive and negative recommendations, as well as different health care services types. We focused particularly on outpatient primary health care services, as they are relevant to the biggest part of the population. However, we also included some discouraged services outside primary health care to extend the spectrum of populations investigated.

The final selection comprised 24 services, including services for screening (N=4), diagnosis (N=6), primary prevention (N=1), treatment (N=4), and secondary prevention (N=9). Definitions of the selected services are provided in Supplementary file 1.

For peer review only

For peer review only - http://bmjopen.bmj.com/site/about/guidelines.xhtml

Assessment of recommendations: strength and direction

Once the services were selected, their associated recommendations were formally assessed.

For each service, we selected the guideline in which the service was originally identified, and also looked up corresponding guidelines by the relevant European, American, and international clinical societies. From this set of guidelines, we selected for assessment the one that was the most applicable to Switzerland in 2014 (see Supplementary file 2 for the prioritization algorithm). We did not consider the guidelines of Swiss medical societies, as their quality of reporting is partially low [19], and they tend to be consistent with European and international guidelines. If the service was initially selected based on a guideline published after 2014, and no applicable guideline could be identified for 2014, the recommendation was automatically considered weak.

Thus, a single recommendation statement was assessed for each service. The assessment was done in duplicate by two authors (AU and HD, both medical doctors). Discordant judgements were resolved with mutual agreement in a discussion. Each recommendation was classified as strong or weak (corresponding to GRADE definition [20]), and positive or negative. The algorithm and criteria for the classification are detailed in Supplementary file 2 for the strength, and in Supplementary file 3 for the direction of a recommendation. The list of guidelines containing the recommendation statements that were assessed is provided in Supplementary file 4.

Swiss health insurance claims data

The utilization of the selected health care services was evaluated using mandatory health insurance claims data from the Helsana Group, covering approximately 1.2 million people (15% of the Swiss population). Helsana Group is one of several private companies providing mandatory health insurance in Switzerland. Eligible patient populations were identified from the patients enrolled with Helsana in 2014. Patients with incomplete address information, living in nursing homes and receiving reimbursement via lump-sums (masking some outpatient services), asylum seekers, those living outside Switzerland, and Helsana employees were excluded.

The data provided by Helsana were anonymized. According to the national ethical and legal regulations, ethical approval was not needed for this type of analysis. This was confirmed by a waiver of the competent ethics committee (Kantonale Ethikkommission Zürich, dated January 11, 2017, BASEC-Nr. Req-2017-00011).

For peer review only - http://bmjopen.bmj.com/site/about/guidelines.xhtml

Models of geographic variation

The utilization of each health care service was determined for each member of the eligible population (see Supplementary file 1 for definitions of the populations and services). For each service, the resulting binary outcome variable was modelled with a multilevel logistic regression technique, using 106 Swiss MobSpat regions (“mobilité spatiale”), as defined by the Swiss Federal Statistical Office [21], as the higher level. Each study participant’s residence was assigned to the corresponding MobSpat region.

Fixed effects were estimated for the following explanatory variables: age, sex, number of comorbidities (0, 1, 2, and 3 or more), and clinical characteristics of relevance for specific indicators (see Supplementary file 1). These variables are often viewed as associated with *warranted* variation [22]. In contrast, we did not adjust for variables associated with *unwarranted* variation (e.g., insurance characteristics or provider density). From each multilevel model, we extracted the variance of the regional random effects, reflecting the potentially unwarranted geographic variation. We also converted the variance to median odds ratios (MORs) for more convenient interpretation [23, 24] and plotting. MOR is interpreted as the median odds of service utilization by two individuals with identical characteristics in two randomly selected regions. As MOR is directly extrapolated from the variance, the ranking of these two parameters coincides.

For peer review only

Statistical analysis of the hypotheses

Variances of the regional random effects of services utilization from the models were used as data points in the final analysis of the hypotheses. Variances of services associated with weak and strong recommendations, as well as negative and positive recommendations were compared with Welch’s unequal variances t-test. Mean differences and 95% confidence intervals were presented. The same analysis was also performed for MOR, to improve interpretability of detected group differences.

Although the number of the services analysed was rather small (24), the distribution of the analysed variances was deemed sufficiently close to normal to warrant the use of parametric tests. To account for the small and unequal sample sizes, we used Welch’s t-test, which is considered more robust in this setting [25]. Confidence intervals were not adjusted for multiple testing.

For peer review only - http://bmjopen.bmj.com/site/about/guidelines.xhtml

Statistical analyses were performed using R 3.6.0 [26] and MLwiN 3.01 [27] integrated in STATA 14.2 using the runmlwin package [28].

Patient and public involvement

This study was performed as part of the National Research Programme 74 “Smarter Health Care” of the Swiss National Science Foundations. Patients and public, including policy makers and healthcare services providers, are involved in interpreting, disseminating and translating the overall results of studies conducted under this programme. Representatives of patients, health care providers, health insurers, and health care policy makers are members of the advisory board of the project. They provided feedback on the planned study design and its preliminary results. Individual patients were not directly involved in the planning and conducting of this study.

Results

Characteristics of the eligible populations and the geographic variation of the services are shown in Table 1. Across the services, the sizes of the eligible populations ranged from 1 992 patients with a new disease-modifying antirheumatic drug (DMARD) prescription to 409 960 patients with recommended influenza vaccination. MOR, reflecting potentially unwarranted geographic variation in utilization of the services, ranged from 1.13 [1.02-1.29] for aspirin in secondary prevention of myocardial infarction (MI) to 1.68 [1.53-1.87] for minor surgical procedures performed in inpatient instead of outpatient settings.

For peer review only - http://bmjopen.bmj.com/site/about/guidelines.xhtml

Table 1 Characteristics of the recommended or discouraged health care services studied

Category	Health care service (abbreviated)	Utilization in eligible population	Eligible population			Recommendation		Random effects in multilevel model	
			Total N	Mean age (sd)	Female N (%)	Strength	Direction	Variance	Median odds ratio (MOR)
Screening	Colon cancer screening	5.9%	276387	58.6 (5.8)	142675 (51.6%)	Strong	Positive	0.04[0.03-0.06]	1.21[1.17-1.26]
	Breast cancer screening	20.9%	178145	61.0 (7.2)	178145 (100%)	Weak	Positive	0.22[0.16-0.29]	1.56[1.47-1.67]
	Prostate cancer screening	28.4%	145874	59.1 (6.2)	0 (0%)	Weak	Positive	0.07[0.05-0.10]	1.29[1.25-1.35]
	Osteoporosis screening	4.9%	60812	72.6 (8.7)	60812 (100%)	Weak	Positive	0.08[0.04-0.13]	1.31[1.22-1.41]
Diagnosis	DM: HbA1c test	69.6%	49198	66.6 (13.0)	22138 (45.0%)	Strong	Positive	0.17[0.12-0.23]	1.48[1.40-1.58]
	DM: renal function test	44.3%	49198	66.6 (13.0)	22138 (45.0%)	Strong	Positive	0.06[0.04-0.09]	1.27[1.22-1.33]
	DM: LDL test	44.3%	33975	60.1 (11.2)	13977 (41.2%)	Strong	Positive	0.13[0.09-0.19]	1.42[1.34-1.51]
	DM: eye examination	55.5%	49198	66.6 (13.0)	22138 (45.0%)	Weak	Positive	0.07[0.05-0.10]	1.29[1.24-1.35]
	**TSH screening**	76.1%	169232	56.8 (18.5)	111847 (66.1%)	Strong	Negative	0.18[0.13-0.25]	1.50[1.42-1.61]
	**POCR**	13.0%	47215	60.3 (17.2)	27086 (57.4%)	Strong	Negative	0.18[0.13-0.26]	1.50[1.40-1.62]
Primary prevention	Influenza vaccination	20.9%	409960	64.1 (16.3)	230202 (56.2%)	Strong	Positive	0.04[0.03-0.05]	1.20[1.17-1.24]
Treatment	**Benzodiazepines**	18.6%	243951	75.0 (7.6)	141986 (58.2%)	Strong	Negative	0.14[0.10-0.18]	1.42[1.36-1.50]
	**Proton pump inhibitors**	55.5%	153523	55.7 (17.8)	93543 (60.9%)	Weak	Negative	0.02[0.02-0.03]	1.16[1.13-1.19]
	**Inpatient procedures**	61.4%	10656	50.5 (13.7)	7719 (72.4%)	Weak	Negative	0.30[0.20-0.43]	1.68[1.53-1.87]
	**Caesarean section**	28.5%	9449	31.9 (5.1)	9449 (100%)	Weak	Negative	0.05[0.02-0.09]	1.24[1.16-1.34]
Secondary prevention	AMI: aspirin	47.0%	2232	72.4 (13.7)	801 (35.9%)	Strong	Positive	0.02[0.00-0.07]	1.13[1.02-1.29]
	AMI: statin	34.2%	2232	72.4 (13.7)	801 (35.9%)	Strong	Positive	0.14[0.06-0.27]	1.43[1.25-1.63]
	AMI: beta-blocker	42.1%	2232	72.4 (13.7)	801 (35.9%)	Strong	Positive	0.05[0.00-0.13]	1.25[1.05-1.40]
	AMI: ACE/ARB	43.8%	2232	72.4 (13.7)	801 (35.9%)	Strong	Positive	0.04[0.00-0.12]	1.21[1.03-1.39]
	AMI: P2Y12 inhibitors	46.8%	2232	72.4 (13.7)	801 (35.9%)	Strong	Positive	0.03[0.00-0.10]	1.18[1.04-1.36]
	PPI with NSAID	43.5%	95072	61.0 (16.2)	60804 (64.0%)	Strong	Positive	0.02[0.01-0.03]	1.15[1.12-1.18]
	PAD: statin	28.5%	23868	63.6 (16.5)	12113 (50.7%)	Strong	Positive	0.04[0.03-0.07]	1.22[1.17-1.28]
	Afib: anticoagulation	27.5%	8291	80.8 (7.9)	4037 (48.7%)	Strong	Positive	0.05[0.02-0.09]	1.24[1.16-1.33]
	GCC with new DMARD	58.7%	1992	59.2 (15.3)	1369 (68.7%)	Weak	Positive	0.06[0.01-0.18]	1.27[1.07-1.49]

Health care services, highlighted in bold, are associated with a negative recommendation.

For peer review only

For peer review only - http://bmjopen.bmj.com/site/about/guidelines.xhtml

SD – standard deviation, N – number, DM – diabetes mellitus, LDL – low density lipid, TSH – thyroid-stimulating hormone, POCR – preoperative chest radiography, AMI – acute myocardial infarction, ACE/ARB – angiotensin-converting enzyme inhibitors or angiotensin II receptor blockers, PPI – proton pump inhibitors, NSAID – nonsteroidal anti-inflammatory drugs, PAD – peripheral artery disease, Afib – atrial fibrillation, GCC – glucocorticosteroid drugs, DMARD – disease-modifying antirheumatic drug.

For peer review only

For three services, a major guideline relevant in 2014 in Switzerland could not be identified (long-term use of proton pump inhibitors, minor inpatient surgery procedures, elective Caesarean section). A total of eight services had weak, and six services had negative underlying recommendations. Median MOR was 1.29 for services with weak and 1.25 for services with strong recommendations; 1.26 for services with positive and 1.46 for services with negative recommendations (Figure 1).

Figure 1 Geographic variation of the health care services grouped by strength (A) and direction (B) of recommendations

A Weak and strong recommendations; B Positive and negative recommendations. MOR – median odds ratio. Boxplots depict the interquartile range of values (upper and lower hinges), and the median value.

Based on Welch's t-test, the difference in mean variances [95CI%] of services with weak and strong recommendations was 0.03 [-0.06, 0.11], and the difference in mean MOR was 0.05 [-0.11, 0.21]. The difference in mean variances [95CI%] of services with negative and positive recommendations was 0.07 [-0.03, 0.18], and the difference in mean MOR was 0.14 [-0.06, 0.34].

Discussion

We did not find a direct association between the strength of clinical recommendation and the geographic variation in the utilization of 24 health care services. The geographic variation in the utilization of services with underlying negative recommendations was slightly higher than for those with positive recommendations. In general, moderate potentially unwarranted geographic variation was observed, with MOR smaller than 1.50 for all but one service.

At least two other studies have to some extent examined the association between the strength of recommendations and the variation in adherence, each focusing on a single clinical specialty. In et al [7], examining a set of recommendations in oncology, found higher variation in the utilization of services associated with a lower level of evidence. However, this study focused not on regional but on inter-institutional variation, comparing two groups of providers. In contrast to this study and in agreement with our results, Mayer et al [8]

For peer review only

found that surgeon practices in knee and hip arthroplasty in Australia varied regardless of the strength of evidence available. Potentially, different clinical areas could be associated with different barriers to guideline implementation, modifying the relationship between recommendations and variation.

To better understand why a direct association of recommendation strength and variation in adherence was not observed, it may be useful to revisit the EBM framework [1]. The EBM framework is *normative* and defines how clinical decisions *should* be made [1, 29]. However, this may not always coincide with how decisions *are* made – a process analysed by *descriptive* theories [29]. In fact, the EBM model has been developed as a conceptual rather than practical guidance of evidence implementation [1], and has not yet generated a coherent theory of clinical decision making, and in particular, of how evidence is incorporated [30]. Thus, although a direct relationship between the strength of recommendation and the geographic variation of service utilization would be encouraged by the normative EBM framework, it may not always be observed.

There are numerous reasons why even strong recommendations [31, 32], or conclusive research evidence more broadly [33], may not directly translate into clinical practice. Research on knowledge translation has identified multiple barriers at different levels of the health care system, including structural, organizational, peer-group, and professional factors [34] – many of which depend on the specific context where a service is provided, and thus may vary geographically. Knowledge transfer processes are highly non-linear and rely on triggering mechanisms [35]. Factors external to research evidence significantly affect translation – potentially creating large geographic heterogeneity even within services with strong recommendations.

An influential framework, explaining different degrees of variation between health care services, has been proposed by Wennberg and colleagues [36]. According to this framework, services are classified into effective, preference-sensitive, and supply-sensitive care. Effective care (services based on solid evidence, so that virtually all patients would choose them) largely corresponds to services with strong recommendations, as defined by GRADE and applied in this study [2]. Preference-sensitive care partly corresponds to services with weak recommendations, as they both imply trade-offs of risks and benefits of multiple options of care [36]. The utilization of preference-sensitive surgical procedures usually has higher variation than of those associated with effective care [37]. In contrast, supply-sensitive care defines the frequency, setting, and intensity of care provision rather than specific types of health care services. It is associated with high, supply-related variation,

For peer review only

but is rarely discussed in guidelines [37], and therefore, could not be included in our study. However, the service of minor surgeries performed as inpatient instead of outpatient procedures could be considered close to the supply-sensitive category. In fact, it had the highest MOR (1.68) in our study.

Regarding the secondary hypothesis, we found that services associated with negative recommendations had slightly higher geographic variation. We did not find other studies directly comparing the regional variation of services with the direction of recommendations. Few studies, focusing mostly on low-value care, have reported MOR as an expression of geographic variation, further limiting the comparison. For example, in a study by Badgery-Parker et al [38], services discouraged by *Choosing Wisely* were shown to have regional MOR from 1.1 to 2.6 – a range that includes all of our observed MORs.

Negative recommendations usually address a widespread service that lacks supporting evidence of benefit or the benefit is outweighed by harms [2]. In contrast to services with positive recommendations, which are introduced after supporting evidence is produced, services with negative recommendations typically become part of the clinical practice *before* evidence is sufficient to rule out their overall benefit. Therefore, their use could be related more to clinical expertise and practice, and could be expected to vary locally. Indeed, the barriers to implementing positive and negative recommendations seem to be different [15] – signalling that the pathways how they are interpreted and integrated into clinical decisions might also be different. As Choosing Wisely and similar initiatives are increasingly gaining attention [39], our finding of higher geographic variation associated with negative recommendations may inform future research and implementation strategies.

This exploratory study has several limitations. First, although we aimed at a balanced selection of clinical fields and service types, the number (24) and range of studied services was limited by the data source. Swiss claims data lack information on outpatient diagnoses, inpatient treatment details, and clinical information such as test results [18]. Lack of clinical information also meant that some populations were not as specific as defined by the recommendation. For example, beta-blockers and angiotensin-converting-enzyme (ACE) inhibitors are recommended after a myocardial infarction contingent on heart failure and left ventricular dysfunction. As such clinical details were unavailable, we had to rely on them being present in the majority of the hospitalized myocardial infarction cases and distributed equally geographically. However, we believe that estimates of variation are accurate, as each of the 24 data points was generated by multilevel modelling of utilization in

For peer review only - http://bmjopen.bmj.com/site/about/guidelines.xhtml

populations of 85000 patients on average, including all major explanatory variables such as age, sex, and indicators of morbidity. Second, the services studied were unavoidably different by characteristics other than the strength or direction of recommendation, such as service type or clinical area, potentially resulting in confounding. Third, both the observed utilization and its geographic variation depend on the definition of the service and population [40]. We aimed to measure the *unwarranted* variation in utilization by using service-specific denominators (eligible populations) and adjusting for relevant clinical characteristics. How exactly *unwarranted* and *warranted* variation should be defined and measured, and what adjustments are necessary to differentiate them, is debated [22, 41]. Fourth, the grouping of recommendations by strength and direction was partly subjective, although we tried to make it reproducible with a clear algorithm, implemented in duplicate. Unfortunately, many different systems for evaluating the strength of recommendations exist [42], which cannot be easily reconciled, and the most prominent, GRADE approach, is not always explicitly used.

To explore the studied questions further, the sample of services could be expanded to inpatient and specialist care. Further, a meta-study of the numerous individual studies of geographic variation in health care services could be undertaken. However, this would currently be challenging, as studies choose different adjustment variables and specificity of studied populations, and report the variation in different quantitative forms (e.g., MOR, systematic component of variation, range). Furthermore, there is a need for qualitative studies of the reasons for the variability of clinical decisions, and how clinical expertise in these decisions interacts with evidence, clinical circumstances, and patient preferences. Qualitative evidence could help to generate more complex hypotheses for further quantitative studies, built on a finer understanding of all the factors influencing the variability of clinical decisions. Specialty-specific sets of services could also be further investigated.

Conclusions

In this exploratory study of 24 health care services mostly in the outpatient primary care setting, we did not observe a significant difference in the degree of geographic variation in utilization of services associated with strong versus weak recommendations. Services associated with negative recommendations had slightly higher geographic variation. The relationship between the strength of recommendations and the variation may be

For peer review only - http://bmjopen.bmj.com/site/about/guidelines.xhtml
For peer review only

indirect or significantly modified by other characteristics of services, such as service type or clinical area. As initiatives discouraging low-value care are gaining attention worldwide, these findings may inform future research in this area.

Funding

This work was supported by the Swiss National Science Foundation (SNSF) National Research Program "Smarter Health Care" (NRP 74), as part of project number 26, grant number 407440_167349.

Competing interests

MS declares a grant from Helsana Insurance Group, outside the submitted work. Helsana Group provided support in the form of salaries for authors BB, EB and CB, but did not have any additional role in the study design, data collection and analysis, decision to publish, or preparation of the manuscript. The other authors declare no competing interests.

Authors' contributions

MS, VvW and HD developed the underlying study program. AU and HD developed study design, with support from all authors. AU, WW, CB, EB, BB did data extraction, preparation and management. WW, MS and OG developed multilevel models applied in this study. AU and WW analysed the data. AU drafted the manuscript, with major inputs from HD and MS, and contributions from all authors. All authors read and approved the final manuscript.

Data sharing statement

The data underlying this study cannot be shared publicly because they are the property of Helsana (https://www.helsana.ch/en/helsana-group), and have restricted public access on grounds of patient privacy. The data are managed by Helsana and subsets of the database are available for researchers after request and under specific conditions. Data are available from Helsana (gesundheitskompetenz@helsana.ch) for researchers who meet the criteria for access to confidential data. Helsana will consider the possibilities of the research

For peer review only

For peer review only - http://bmjopen.bmj.com/site/about/guidelines.xhtml

proposal and decide to grant access if the research questions can be answered with use of the Helsana data. When requests are granted, data are accessible only in a secure environment.

References

1. Haynes RB, Devereaux PJ, Guyatt GH. Clinical expertise in the era of evidence-based medicine and patient choice. Evid Based Med. 2002;7:36–8.

2. Andrews J, Guyatt G, Oxman AD, Alderson P, Dahm P, Falck-Ytter Y, et al. GRADE guidelines: 14. Going from evidence to recommendations: The significance and presentation of recommendations. J Clin Epidemiol. 2013;66:719–25. doi:10.1016/j.jclinepi.2012.03.013.

3. Andrews JC, Schünemann HJ, Oxman AD, Pottie K, Meerpohl JJ, Coello PA, et al. GRADE guidelines: 15. Going from evidence to recommendation - Determinants of a recommendation's direction and strength. J Clin Epidemiol. 2013;66:726–35. doi:10.1016/j.jclinepi.2013.02.003.

4. Cutler D, Skinner JS, Stern AD, Wennberg D. Physician beliefs and patient preferences: A new look at regional variation in health care spending. Am Econ J Econ Policy. 2019;11:192–221.

5. Finkelstein A, Gentzkow M, Williams H. Sources of Geographic Variation in Health Care: Evidence From PatientMigration. Q J Econ. 2016;131:1681–726.

6. Molitor D. The evolution of physician practice styles: Evidence from cardiologist migration. Am Econ J Econ Policy. 2018;10:326–56.

7. In H, Neville BA, Lipsitz SR, Corso KA, Weeks JC, Greenberg CC. The role of National Cancer Institute-designated cancer center status: observed variation in surgical care depends on the level of evidence. Ann Surg. 2012;255:890–5. doi:10.1097/SLA.0b013e31824deae6.

8. Mayer M, Naylor J, Harris I, Badge H, Adie S, Mills K, et al. Evidence base and practice variation in acute care processes for knee and hip arthroplasty surgeries. PLoS One. 2017;12:e0180090. doi:10.1371/journal.pone.0180090.

9. Corallo AN, Croxford R, Goodman DC, Bryan EL, Srivastava D, Stukel TA. A systematic review of medical practice variation in OECD countries. Health Policy (New York). 2014;114:5–14. doi:10.1016/j.healthpol.2013.08.002.

For peer review only

10. The Dartmouth Atlas of Health Care. https://www.dartmouthatlas.org/. Accessed 11 May 2020.

11. Public Health England. Public Health Profiles. https://fingertips.phe.org.uk/profile/atlas-of-variation. Accessed 11 May 2020.

12. Cassel CK, Guest JA. Choosing wisely: Helping physicians and patients make smart decisions about their care. JAMA - Journal of the American Medical Association. 2012;307:1801–2.

13. Elshaug AG, Watt AM, Mundy L, Willis CD. Over 150 potentially low-value health care practices: an Australian study. Med J Aust. 2012;197:556–60.

14. Prasad V, Vandross A, Toomey C, Cheung M, Rho J, Quinn S, et al. A decade of reversal: An analysis of 146 contradicted medical practices. Mayo Clin Proc. 2013;88:790–8.

15. Carlsen B, Glenton C, Pope C. Thou shalt versus thou shalt not: A meta-synthesis of GPs' attitudes to clinical practice guidelines. Br J Gen Pract. 2007;57:971–8.

16. National Research Programme 74. Project 26: How do guidelines and recommendations influence medical treatment? http://www.nfp74.ch/en/projects/healthcare-across-sectors/project-schwenkglenks. Accessed 15 Apr 2020.

17. Swiss Federal Office of Public Health and Swiss Conference of Cantonal Health Directors. Nationale Strategie Prävention nichtübertragbarer Krankheiten (NCD-Strategie) 2017-2024 [National Strategy of Non-communicable Diseases]. 2016.

18. Ulyte A, Bähler C, Schwenkglenks M, von Wyl V, Gruebner O, Wei W, et al. Measuring diabetes guideline adherence with claims data: systematic construction of indicators and related challenges. BMJ Open. 2019;9:e027138. doi:10.1136/bmjopen-2018-027138.

19. Bachmann L, Ulytė A, Dressel H. Clinical practice guidelines of medical societies in Switzerland: analysis of the current state. Swiss Med Wkly. 2019;149:w20134.

20. Guyatt GH, Oxman AD, Kunz R, Falck-Ytter Y, Vist GE, Liberati A, et al. GRADE: Going from Evidence to Recommendations. BMJ. 2008;336:1048–51.

21. Bundesamt für Statistik. MS-Regionen. https://www.bfs.admin.ch/bfs/de/home/statistiken/raum-umwelt/nomenklaturen/msreg.assetdetail.415729.html. Accessed 11 May 2020.

22. Sutherland K, Levesque J. Unwarranted clinical variation in health care: Definitions and proposal of an

For peer review only

For peer review only - http://bmjopen.bmj.com/site/about/guidelines.xhtml

analytic framework. J Eval Clin Pract. 2019;:jep.13181. doi:10.1111/jep.13181.

23. Larsen K, Petersen JH, Budtz-Jørgensen E, Endahl L. Interpreting parameters in the logistic regression model with random effects. Biometrics. 2000;56:909–14.

24. Larsen K, Merlo J. Appropriate Assessment of Neighborhood Effects on Individual Health: Integrating Random and Fixed Effects in Multilevel Logistic Regression. Am J Epidemiol. 2005;161:81–8.

25. Ruxton GD. The unequal variance t-test is an underused alternative to Student's t-test and the Mann-Whitney U test. Behav Ecol. 2006;17:688–90.

26. R Core Team. R: A language and environment for statistical computing. R Foundation for Statistical Computing, Vienna, Austria. 2019. https://www.r-project.org/. Accessed 26 Nov 2019.

27. Charlton, C, Rasbash, J, Browne, WJ, Healy, M, Cameron B. MLwiN Version 3.01. Centre for Multilevel Modelling, University of Bristol. 2017.

28. Leckie G, Charlton C. Runmlwin: A program to run the MLwiN multilevel modeling software from within Stata. J Stat Softw. 2013;52:1–40.

29. Djulbegovic B, Elqayam S, Dale W. Rational decision making in medicine: Implications for overuse and underuse. J Eval Clin Pract. 2018;24:655–65. doi:10.1111/jep.12851.

30. Djulbegovic B, Guyatt GH. Progress in evidence-based medicine: a quarter century on. The Lancet. 2017;390:415–23.

31. Francke AL, Smit MC, De Veer AJE, Mistiaen P. Factors influencing the implementation of clinical guidelines for health care professionals: A systematic meta-review. BMC Med Inform Decis Mak. 2008;8:38. doi:10.1186/1472-6947-8-38.

32. Lugtenberg M, Zegers-Van Schaick JM, Westert GP, Burgers JS. Why don't physicians adhere to guideline recommendations in practice? An analysis of barriers among Dutch general practitioners. Implement Sci. 2009;4:1–9.

33. Wallace J, Nwosu B, Clarke M. Barriers to the uptake of evidence from systematic reviews and meta-analyses: A systematic review of decision makers' perceptions. BMJ Open. 2012;2:e001220.

34. Grimshaw JM, Eccles MP, Lavis JN, Hill SJ, Squires JE. Knowledge translation of research findings. Implement Sci. 2012;7:50. doi:10.1186/1748-5908-7-50.

For peer review only

For peer review only - http://bmjopen.bmj.com/site/about/guidelines.xhtml

35. Braithwaite J, Churruca K, Long JC, Ellis LA, Herkes J. When complexity science meets implementation science: A theoretical and empirical analysis of systems change. BMC Med. 2018;16:63. doi:10.1186/s12916-018-1057-z.

36. Wennberg JE, Fisher ES, Skinner JS. Geography and the debate over medicare reform. Health Affairs. 2003;22 SUPPL.

37. Wennberg JE. Tracking medicine : a researcher’s quest to understand health care. Oxford University Press; 2010.

38. Badgery-Parker T, Feng Y, Pearson S-A, Levesque J-F, Dunn S, Elshaug AG. Exploring variation in low-value care: a multilevel modelling study. BMC Health Serv Res. 2019;19:345.

39. Levinson W, Kallewaard M, Bhatia RS, Wolfson D, Shortt S, Kerr E a. “Choosing Wisely”: a growing international campaign. BMJ Qual Saf. 2015;24:167–74. doi:10.1136/bmjqs-2014-003821.

40. Schwartz AL, Landon BE, Elshaug AG, Chernew ME, McWilliams JM. Measuring low-value care in Medicare. JAMA Intern Med. 2014;174:1067–76. doi:10.1001/jamainternmed.2014.1541.

41. Harrison R, Manias E, Mears S, Heslop D, Hinchcliff R, Hay L. Addressing unwarranted clinical variation: A rapid review of current evidence. J Eval Clin Pract. 2019;25:53–65. doi:10.1111/jep.12930.

42. Atkins D, Eccles M, Flottorp S, Guyatt GH, Henry D, Hill S, et al. Systems for grading the quality of evidence and the strength of recommendations I: Critical appraisal of existing approaches. BMC Health Serv Res. 2004;4:1–7.

For peer review only

Supplementary files

Supplementary file 1 Definitions and descriptions of the studied health care services and eligible populations

Supplementary file 2 Algorithm and criteria for the assessment of the strength of recommendation

Supplementary file 3 Algorithm and criteria for the assessment of the direction of recommendation

Supplementary file 4 List of guidelines selected for the study, describing the services analysed

For peer review only - http://bmjopen.bmj.com/site/about/guidelines.xhtml

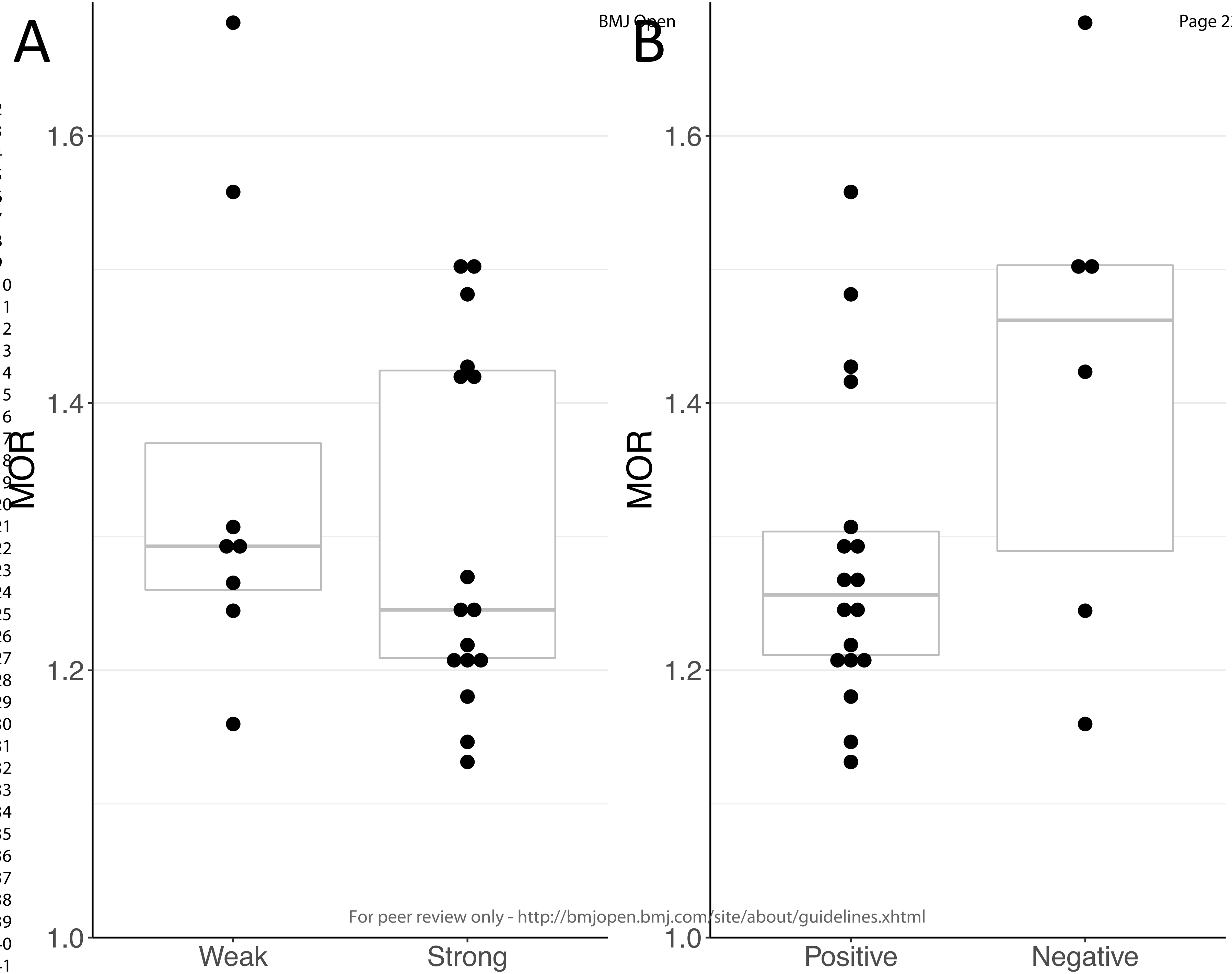
A
B
MOR
1.0
1.2
1.4
1.6
Weak
Strong
Positive
Negative

Additional file 1 Definitions and descriptions of the studied health care services and eligible populations

**Category**	**Health care service**	**Service description and frequency**	**Eligible population**	**Recommendation**	**Specific clinical explanatory variables**
Screening	Colon cancer screening	Colonoscopy/ year	Anyone 50-69 years old	Colonoscopy should be done every 10 years for people 50-69 years old.	Previous treatment of cancer or inflammatory bowel disease, hospitalization with colon disease in the last year
	Breast cancer screening	Mammography/ year	50-74 years old women	Mammography should be done every 2 years for 50-74 years old women.	Previous treatment of breast or other cancer
	Prostate cancer screening	Prostate-specific antigen (PSA) testing/ year	50-70 years old men	Early detection of prostate cancer (opportunistic screening) should be offered to the well-informed man.	Previous treatment of cancer, hospitalization with prostate disease in the last year
	Osteoporosis screening	Dual-energy x-ray absorptiometry (DXA)/ year	Women over 60 and with risk factors[a] of spontaneous fractures	DXA densitometry is recommended for postmenopausal women with spontaneous fractures or increased risk of them.	Presence of more than one risk factor
Diagnosis	DM: HbA1c test	Glycated haemoglobin (HbA1c) test twice/ year	Adult drug-treated diabetes patients	HbA1c test should be done for diabetes patients at least twice a year.	Oral diabetes medication or insulin
	DM: renal function test	Albuminuria and serum creatinine tests/ year	Adult drug-treated diabetes patients	Albuminuria and serum creatinine tests should be done for diabetes patients at least once a year.	Oral diabetes medication or insulin
	DM: LDL test	Low-density lipoprotein (LDL) test/ year	Adult drug-treated diabetes patients under 75 years old	LDL test should be done for diabetes patients at least once a year.	Oral diabetes medication or insulin

For peer review only

	DM: eye examination	Ophthalmologist visit/ year	Adult drug-treated diabetes patients	Eye exam should be performed for diabetes patients at least once a year.	Oral diabetes medication or insulin
	TSH screening	Thyroid-stimulating hormone (TSH) test without T3 and T4 tests on the same day	Adults without thyroid disease and receiving TSH test	TSH should be measured as an initial screening test for hypo/hyperthyroidism, while T3 and T4 test should follow if TSH is abnormal.	-
	POCR	Outpatient preoperative chest radiography (POCR) up to 2 months before surgery	Adult patients with inpatient surgical procedures	Routine chest radiography is not recommended before surgery.	-
Primary prevention	Influenza vaccination	Influenza outpatient vaccination/ year	People over 65 years old or with a specified chronic condition[b]	People over 65 years old and patients with chronic conditions, specified by Federal Office of Public Health, should be vaccinated against influenza every year.	Hospitalization with pneumonia in the last year
Treatment	Benzodiazepines	Cumulative prescription of benzodiazepines (BZD) for >8 weeks/ year	Anyone over 65 years old	Long-term use of benzodiazepines and other hypnotics is discouraged for old patients.	Treated epilepsy, stay in a nursing home in the last year, hospitalization in the last year with a diagnosis indicative of justified benzodiazepine use
	Proton pump inhibitors	Cumulative prescription of proton pump inhibitors (PPI) or H2 histamine receptor antagonists (H2) for >8 weeks/ year	Adults receiving PPI or H2 drugs	PPI should not be used at maximal dose for prolonged periods of time.	-

For peer review only - http://bmjopen.bmj.com/site/about/guidelines.xhtml

	Inpatient procedures	Specified surgical procedures[c] done in the outpatient setting	Adult patients with specified surgical procedures (either as in- or outpatient)	If none of the special conditions apply, certain surgical procedures should be done in the outpatient setting.	-
	Caesarean section	Caesarean section (C-section)	Women giving birth without absolute indications[d] for C-section	C-section should not be performed unless absolute or relative indications are present.	-
Secondary prevention	AMI: aspirin	Aspirin prescription within 2 weeks after acute myocardial infarction (AMI)	Adult patients with AMI	All myocardial infarction patients should take aspirin long-term.	Hospitalization for stroke or bleeding event or prescribed anticoagulation in the last year
	AMI: statin	High-dose statin prescription within 2 weeks after AMI	Adult patients with AMI	All myocardial infarction patients should get statins long-term.	Hospitalization for stroke in the last year
	AMI: beta-blocker	Beta-blocker prescription within 2 weeks after AMI	Adult patients with AMI	All myocardial infarction patients with heart failure or impaired function should get beta-blockers long-term.	Hospitalization with heart failure diagnosis in the last year
	AMI: ACE/ARB	Angiotensin-converting enzyme (ACE) or angiotensin receptor blocker (ARB) antihypertensive medication prescription within 2 weeks after AMI	Adult patients with AMI	All myocardial infarction patients with heart failure or impaired function should get ACE or ARB antihypertensive medication long-term.	-

For peer review only

	AMI: P2Y12 inhibitors	P2Y12 antiplatelet drug[e] prescription within 2 weeks after AMI	Adult patients with AMI	All myocardial infarction patients should get P2Y12 antiplatelet drugs for at least 1-12 months according to the bleeding risk profile and AMI treatment.	Hospitalization for a bleeding event or prescribed anticoagulation in the last year
	PPI with NSAID	PPI prescription within 1 month or up to 3 months before initial long-term nonsteroidal anti-inflammatory drug (NSAID) prescription	Adult patients with a cumulative NSAID prescription of >8 weeks at maximal dose	Patients taking long-term NSAID and with risk factors for gastric ulcer[f] should also take PPI.	Concurrent use of antiplatelet, anticoagulation drugs or oral glucocorticoids, hospitalization for bleeding event in the last year.
	PAD: statin	Prescription of statins within 3 months after peripheral artery disease (PAD) identification	Adult patients undergoing diagnostic or treatment procedures for PAD	Statins are recommended for all patients with PAD.	-
	Afib: anticoagulation	Oral anticoagulation prescription within 2 weeks after atrial fibrillation (Afib) identification	Adult patients with atrial fibrillation diagnosis and additional risk factors[g]	All patients with atrial fibrillation should be prescribed oral anticoagulation for embolic events prevention according to the CHA_2DS_2-VASc score.	-
	GCC with new DMARD	Glucocorticoid (GCC) prescription within 1 month or up to 3 months before disease-modifying antirheumatic drug (DMARD) prescription	Adult patients with a new prescription of DMARD by a rheumatologist	Short-term glucocorticoids should be taken with newly prescribed DMARD.	-

a. Recent distal radius, proximal humerus, vertebral or femoral fracture, use of drugs increasing the risk of osteoporosis, use of oral glucocorticoids, diabetes, ankylosing spondylitis, osteogenesis imperfecta, rheumatoid arthritis, inflammatory bowel disease, Cushing's disease, alcohol or nicotine abuse, chronic liver disease, gastrectomy, malnutrition, hypogonadism, hyper- or hypothyroidism, and hyperparathyroidism. Patients currently treated or diagnosed with osteoporosis were excluded.

For peer review only - http://bmjopen.bmj.com/site/about/guidelines.xhtml

b. Cardiovascular disease, chronic pulmonary disease, diabetes, chronic liver disease, renal failure, immune deficiency, systemic neurologic disorders.

c. Varicose veins ligation and stripping, surgical procedures of haemorrhoids, inguinal hernia and cervix, knee arthroscopy and meniscectomy, tonsillectomy.

d. Placental, umbilical cord or fetal pathology, HIV or genital HSV infection, or multiple pregnancy.

e. Clopidogrel, prasugrel or ticagrelor.

f. Concurrent use of antiplatelet, anticoagulant drugs, oral glucocorticoids or recent hospitalization with any major bleeding.

g. Risk factors (congestive heart failure, hypertension, age 65-74 or ≥75 years old, diabetes, previous stroke, transient ischemic attack, or thromboembolism, cardiovascular disease, female sex) were extracted from available claims data and summed according to CHA2DS2-VASc score. Patients with CHA2DS2-VASc score of ≥2 for males and ≥3 for females were included.

DM – diabetes mellitus, HbA1c – Glycated haemoglobin, LDL – low density lipid, TSH – thyroid-stimulating hormone, T3 and T4 – triiodothyronine and thyroxine, POCR – preoperative chest radiography, BZD – benzodiazepines, PPI – proton pump inhibitors, H2 – H2 histamine receptor antagonists, C-section – Caesarean section, AMI – acute myocardial infarction, ACE/ARB – angiotensin-converting enzyme inhibitors or angiotensin II receptor blockers, NSAID – nonsteroidal anti-inflammatory drugs, PAD – peripheral artery disease, Afib – atrial fibrillation, GCC – glucocorticosteroid drugs, DMARD – disease-modifying antirheumatic drug.

For peer review only

Additional file 2 Algorithm and criteria for the assessment of the strength of recommendation

N of authors	Steps
Single	1. Identify the relevant medical societies for each selected health care service.<br>2. Look up the European or international medical societies' websites and journals, and identify relevant guidelines published before 2014. In addition, look up if Swiss federal legislation guidelines exist by 2014.<br>3. If none found, look up American medical society and identify relevant guidelines published before 2014.<br>4. If none found, consider the recommendation **weak**.
In duplicate	5. Once the guideline and the recommendation statement are located, classify the recommendation into strong or weak[a].<br>- **Strong recommendation** implies that the desirable effects of adherence to a recommendation outweigh the undesirable effects.<br>- That means that most informed patients would choose the recommended management and that clinicians can structure their interactions with patients accordingly.<br>- For clinicians, that would mean that most patients should receive the recommended course of action.<br>For patients, that would mean that most people in such a situation would want the recommended course of action and only a small proportion would not; patients should request discussion if the intervention is not offered.<br>- **Weak recommendation** implies that the desirable effects of adherence to a recommendation probably outweigh the undesirable effects, but the guideline panel is less confident.<br>- Thus, a weak recommendation is conditional or optional, and means that patients' choices will vary according to their values and preferences, and clinicians must ensure that patients' care is in keeping with their values and preferences.<br>- For clinicians, that would mean that they should recognize that different choices will be appropriate for different patients and that they must help each patient to arrive at a management decision consistent with her or his values and preferences.<br>For patients, that would mean that most people in such situation would want the recommended course of action, but many would not.

a adapted from: Guyatt GH, Oxman AD, Kunz R, Falck-Ytter Y, Vist GE, Liberati A, et al. GRADE: Going from Evidence to Recommendations. BMJ 2008;336:1048–51.

For peer review only

Additional file 3 Algorithm and criteria for the assessment of the direction of recommendation

N of authors	Steps	
In duplicate	1.	Once the guideline and the recommendation statement are located (see Additional file 2), classify the recommendation into positive and negative.
	-	**Positive** recommendation encourages the use of a health care service in a given population.
	-	**Negative** recommendation discourages the use of a health care service in a given population (e.g., contains negative indicatory words, such as *not, no, never*)

For peer review only

Additional file 4 List of guidelines selected for the study, describing the services analysed

Recommendation	Reference	Comment
Colon cancer screening	[1]	
Breast cancer screening	[2]	
Prostate cancer screening	[3]	
Osteoporosis screening	[4]	
DM: HbA1c test	[5]	
DM: renal function test	[5]	
DM: LDL test	[5]	
DM: eye examination	[5]	
TSH screening	[6,7]	
POCR	[8]	
Influenza vaccination	[9]	
Benzodiazepines	[10]	
Proton pump inhibitors	-	Swiss national guideline since 2016 [11]
Inpatient procedures	-	Swiss federal regulation exists from 2019 [12]
Caesarean section	-	Swiss national guideline since 2015 [13]
AMI: aspirin	[14,15]	
AMI: statin	[14,15]	
AMI: beta-blocker	[14,15]	
AMI: ACE/ARB	[14,15]	
AMI: P2Y12 inhibitors	[14,15]	
PPI with NSAID	[16]	
PAD: statin	[17]	
Afib: anticoagulation	[18]	
GCC with new DMARD	[19]	

DM – diabetes mellitus, HbA1c – Glycated haemoglobin, LDL – low density lipid, TSH – thyroid-stimulating hormone, POCR – preoperative chest radiography, AMI – acute myocardial infarction, ACE/ARB – angiotensin-converting enzyme inhibitors or angiotensin II receptor blockers, PPI – proton pump inhibitors, NSAID – nonsteroidal anti-inflammatory drugs, PAD – peripheral artery disease, Afib – atrial fibrillation, GCC – glucocorticosteroid drugs, DMARD – disease-modifying antirheumatic drug.

1. Lansdorp-Vogelaar I, Karsa L. European guidelines for quality assurance in colorectal cancer screening and diagnosis. First Edition – Introduction. Endoscopy. 2012 Sep 25;44(S 03):SE15–30.
2. Screening for Breast Cancer: U.S. Preventive Services Task Force Recommendation Statement. Ann Intern Med. 2009 Nov 17;151(10):716.
3. Heidenreich A, Bellmunt J, Bolla M, Joniau S, Mason M, Matveev V, et al. EAU Guidelines on Prostate Cancer. Part 1: Screening, Diagnosis, and Treatment of Clinically Localised Disease. Eur Urol. 2011 Jan;59(1):61–71.

For peer review only - http://bmjopen.bmj.com/site/about/guidelines.xhtml

For peer review only

4. Kanis JA, McCloskey E V., Johansson H, Cooper C, Rizzoli R, Reginster J-Y. European guidance for the diagnosis and management of osteoporosis in postmenopausal women. Osteoporos Int. 2013 Jan 19;24(1):23–57.

5. Rydén L, Grant PJ, Anker SD, Berne C, Cosentino F, Danchin N, et al. ESC Guidelines on diabetes, pre-diabetes, and cardiovascular diseases developed in collaboration with the EASD. Eur Heart J. 2013 Oct 14;34(39):3035–87.

6. Bahn RS, Burch HB, Cooper DS, Garber JR, Greenlee MC, Klein I, et al. Hyperthyroidism and Other Causes of Thyrotoxicosis: Management Guidelines of the American Thyroid Association and American Association of Clinical Endocrinologists. Thyroid. 2011 Jun;21(6):593–646.

7. Garber J, Cobin R, Gharib H, Hennessey J, Klein I, Mechanick J, et al. Clinical Practice Guidelines for Hypothyroidism in Adults: Cosponsored by the American Association of Clinical Endocrinologists and the American Thyroid Association. Endocr Pract. 2012 Nov;18(6):988–1028.

8. Choosing Wisely. American College of Surgeons. Admission pre-op chest x-rays. September 4, 2013. Available from: https://www.choosingwisely.org/clinician-lists/american-college-surgeons-admission-or-preop-chest-x-ray-on-ambulatory-patients/ Accessed on May 11, 2020

9. Swiss Federal Office of Public Health. Recommendations for Influenza Vaccination (Empfehlungen zur Grippeimpfung). 2011.

10. Choosing Wisely. American Geriatrics Society. Benzodiazepines sedative hypnotics for insomnia in older adults. February 21, 2013. Available from: https://www.choosingwisely.org/clinician-lists/american-geriatrics-society-benzodiazepines-sedative-hypnotics-for-insomnia-in-older-adults/ Accessed on May 11, 2020

11. Swiss Society of General Internal Medicine. Smarter Medicine. Top-5 List for Ambulatory Care. 2016. Available from: https://www.smartermedicine.ch/de/top-5-listen/ambulante-allgemeine-innere-medizin.html Accessed on May 11, 2020

12. Swiss Federal Office of Public Health. "Outpatient instead of Inpatient" [Änderung der Krankenpflege-Leistungsverordnung (KLV) betreffend «Ambulant vor Stationär»]. Available from: https://www.bag.admin.ch/bag/de/home/versicherungen/krankenversicherung/krankenversicherung-revisionsprojekte/konsultation-ambulant-vor-stationaer.html Accessed on May 11, 2020

13. Hoesli I, Alma-Stucki S El, Drack G, Girard T, Irion O, Schulzke S, et al. Guideline Sectio Caesarea. 2015;1–20. Available from: https://www.sggg.ch/fachthemen/guidelines/ Accessed on May 11, 2020

14. Hamm CW, Bassand J-P, Agewall S, Bax J, Boersma E, Bueno H, et al. ESC Guidelines for the management of acute coronary syndromes in patients presenting without persistent ST-segment elevation: The Task Force for the management of acute coronary syndromes (ACS) in patients presenting without persistent ST-segment elevatio. Eur Heart J. 2011 Dec 1;32(23):2999–3054.

15. Steg PG, James SK, Atar D, Badano LP, Lundqvist CB, Borger MA, et al. ESC Guidelines for the management of acute myocardial infarction in patients presenting with ST-segment elevation. Eur Heart J. 2012 Oct 1;33(20):2569–619.

16. Recommendations for use of selective and nonselective nonsteroidal antiinflammatory drugs: An American College of Rheumatology white paper. Arthritis Rheum. 2008 Aug 15;59(8):1058–73.

For peer review only

17. Tendera M, Aboyans V, Bartelink M-L, Baumgartner I, Clement D, Collet J-P, et al. ESC Guidelines on the diagnosis and treatment of peripheral artery diseases: Document covering atherosclerotic disease of extracranial carotid and vertebral, mesenteric, renal, upper and lower extremity arteries * The Task Force on the Diagnosis and Treat. Eur Heart J. 2011 Nov 2;32(22):2851–906.
18. Camm AJ, Lip GYH, De Caterina R, Savelieva I, Atar D, Hohnloser SH, et al. 2012 focused update of the ESC Guidelines for the management of atrial fibrillation. Eur Heart J. 2012 Nov 1;33(21):2719–47.
19. Smolen JS, Landewé R, Breedveld FC, Buch M, Burmester G, Dougados M, et al. EULAR recommendations for the management of rheumatoid arthritis with synthetic and biological disease-modifying antirheumatic drugs: 2013 update. Ann Rheum Dis. 2014 Mar;73(3):492–509.

For peer review only

STROBE 2007 (v4) Statement—Checklist of items that should be included in reports of *cross-sectional studies*

Section/Topic	Item #	Recommendation	Reported on page #
**Title and abstract**	1	(*a*) Indicate the study's design with a commonly used term in the title or the abstract	1,2
		(*b*) Provide in the abstract an informative and balanced summary of what was done and what was found	2
**Introduction**			
Background/rationale	2	Explain the scientific background and rationale for the investigation being reported	4
Objectives	3	State specific objectives, including any prespecified hypotheses	4,5
**Methods**			
Study design	4	Present key elements of study design early in the paper	2,
Setting	5	Describe the setting, locations, and relevant dates, including periods of recruitment, exposure, follow-up, and data collection	5-7
Participants	6	(*a*) Give the eligibility criteria, and the sources and methods of selection of participants	7, Supplementary file 1
Variables	7	Clearly define all outcomes, exposures, predictors, potential confounders, and effect modifiers. Give diagnostic criteria, if applicable	7, Supplementary file 1
Data sources/ measurement	8*	For each variable of interest, give sources of data and details of methods of assessment (measurement). Describe comparability of assessment methods if there is more than one group	6, Supplementary files 1-4
Bias	9	Describe any efforts to address potential sources of bias	6,7
Study size	10	Explain how the study size was arrived at	5
Quantitative variables	11	Explain how quantitative variables were handled in the analyses. If applicable, describe which groupings were chosen and why	7
Statistical methods	12	(*a*) Describe all statistical methods, including those used to control for confounding	7
		(*b*) Describe any methods used to examine subgroups and interactions	7
		(*c*) Explain how missing data were addressed	NA
		(*d*) If applicable, describe analytical methods taking account of sampling strategy	NA
		(*e*) Describe any sensitivity analyses	NA
**Results**			

For peer review only

Participants	13*	(a) Report numbers of individuals at each stage of study—eg numbers potentially eligible, examined for eligibility, confirmed eligible, included in the study, completing follow-up, and analysed	8,9
		(b) Give reasons for non-participation at each stage	NA
		(c) Consider use of a flow diagram	NA
Descriptive data	14*	(a) Give characteristics of study participants (eg demographic, clinical, social) and information on exposures and potential confounders	9
		(b) Indicate number of participants with missing data for each variable of interest	NA
Outcome data	15*	Report numbers of outcome events or summary measures	9
Main results	16	(*a*) Give unadjusted estimates and, if applicable, confounder-adjusted estimates and their precision (eg, 95% confidence interval). Make clear which confounders were adjusted for and why they were included	9, Supplementary file 1
		(*b*) Report category boundaries when continuous variables were categorized	NA
		(*c*) If relevant, consider translating estimates of relative risk into absolute risk for a meaningful time period	NA
Other analyses	17	Report other analyses done—eg analyses of subgroups and interactions, and sensitivity analyses	NA
**Discussion**			
Key results	18	Summarise key results with reference to study objectives	11
Limitations	19	Discuss limitations of the study, taking into account sources of potential bias or imprecision. Discuss both direction and magnitude of any potential bias	13,14
Interpretation	20	Give a cautious overall interpretation of results considering objectives, limitations, multiplicity of analyses, results from similar studies, and other relevant evidence	14,15
Generalisability	21	Discuss the generalisability (external validity) of the study results	14
**Other information**			
Funding	22	Give the source of funding and the role of the funders for the present study and, if applicable, for the original study on which the present article is based	15

*Give information separately for cases and controls in case-control studies and, if applicable, for exposed and unexposed groups in cohort and cross-sectional studies.

Note: An Explanation and Elaboration article discusses each checklist item and gives methodological background and published examples of transparent reporting. The STROBE checklist is best used in conjunction with this article (freely available on the Web sites of PLoS Medicine at http://www.plosmedicine.org/, Annals of Internal Medicine at http://www.annals.org/, and Epidemiology at http://www.epidem.com/). Information on the STROBE Initiative is available at www.strobe-statement.org.

For peer review only

Are weak or negative clinical recommendations associated with higher geographic variation in utilization than strong or positive recommendations? Cross-sectional study of 24 health care services

Journal:	*BMJ Open*
Manuscript ID	bmjopen-2020-044090.R1
Article Type:	Original research
Date Submitted by the Author:	19-Feb-2021
Complete List of Authors:	Ulyte, Agne; University of Zurich, EBPI<br>Wei, Wenjia; University of Zurich, EBPI<br>Gruebner, Oliver; University of Zurich, EBPI<br>Bähler, Caroline ; Helsana Group, Department of Health Sciences<br>Brüngger, Beat; Helsana Group, Department of Health Sciences<br>Blozik, Eva; Helsana Group, Department of Health Sciences<br>von Wyl, Viktor ; University of Zurich, EBPI<br>Schwenkglenks, M; University of Zurich<br>Dressel, Holger; University of Zurich
**Primary Subject Heading**:	Health services research
Secondary Subject Heading:	Evidence based practice, Health policy, Patient-centred medicine
Keywords:	EPIDEMIOLOGY, Organisation of health services < HEALTH SERVICES ADMINISTRATION & MANAGEMENT, Protocols & guidelines < HEALTH SERVICES ADMINISTRATION & MANAGEMENT, HEALTH SERVICES ADMINISTRATION & MANAGEMENT

SCHOLARONE™
Manuscripts

I, the Submitting Author has the right to grant and does grant on behalf of all authors of the Work (as defined in the below author licence), an exclusive licence and/or a non-exclusive licence for contributions from authors who are: i) UK Crown employees; ii) where BMJ has agreed a CC-BY licence shall apply, and/or iii) in accordance with the terms applicable for US Federal Government officers or employees acting as part of their official duties; on a worldwide, perpetual, irrevocable, royalty-free basis to BMJ Publishing Group Ltd ("BMJ") its licensees and where the relevant Journal is co-owned by BMJ to the co-owners of the Journal, to publish the Work in this journal and any other BMJ products and to exploit all rights, as set out in our licence.

The Submitting Author accepts and understands that any supply made under these terms is made by BMJ to the Submitting Author unless you are acting as an employee on behalf of your employer or a postgraduate student of an affiliated institution which is paying any applicable article publishing charge ("APC") for Open Access articles. Where the Submitting Author wishes to make the Work available on an Open Access basis (and intends to pay the relevant APC), the terms of reuse of such Open Access shall be governed by a Creative Commons licence – details of these licences and which Creative Commons licence will apply to this Work are set out in our licence referred to above.

Other than as permitted in any relevant BMJ Author's Self Archiving Policies, I confirm this Work has not been accepted for publication elsewhere, is not being considered for publication elsewhere and does not duplicate material already published. I confirm all authors consent to publication of this Work and authorise the granting of this licence.

For peer review only

Are weak or negative clinical recommendations associated with higher geographic variation in utilization than strong or positive recommendations? Cross-sectional study of 24 health care services

Authors

Agne Ulyte[a]*, Wenjia Wei[a], Oliver Gruebner[a,b], Caroline Bähler[a,c], Beat Brüngger[a,c], Eva Blozik[c,d], Viktor von Wyl[a], Matthias Schwenkglenks[a], Holger Dressel[e]

Affiliations

a - Department of Epidemiology, Epidemiology, Biostatistics & Prevention Institute, University of Zurich, Hirschengraben 84, 8001 Zurich, Switzerland

b – Department of Geography, University of Zurich, Winterthurerstrasse 190, 8057 Zurich, Switzerland

Hospital Zurich, Hirschengraben 84, 8001 Zurich, Switzerland

c - Department of Health Sciences, Helsana Group, Zürichstrasse 130, 8600 Dübendorf, Switzerland

d - Institute of Primary Care, University of Zurich and University Hospital Zurich, Pestalozzistrasse 24, 8091 Zürich, Switzerland

e – Division of Occupational and Environmental Medicine, Department of Epidemiology, Epidemiology, Biostatistics & Prevention Institute, University of Zurich and University Hospital Zurich, Hirschengraben 84, 8001 Zurich, Switzerland

*corresponding author

Agne Ulyte

Email: agne.ulyte@uzh.ch

Phone: +41 44 63 44681

For peer review only

25 **Address:** University of Zurich, Epidemiology, Biostatistics and Prevention Institute (EBPI), Hirschengraben 84,
26 8001 Zurich, Switzerland

For peer review only

For peer review only - http://bmjopen.bmj.com/site/about/guidelines.xhtml

Abstract

Objectives

When research evidence is lacking, patient and provider preferences, expected to vary geographically, might have a stronger role in clinical decisions. We investigated whether the strength or the direction of recommendation is associated with the degree of geographic variation in utilization.

Design

In this cross-sectional study, we selected 24 services following a comprehensive approach. The strength and direction of recommendations were assessed in duplicate. Multilevel models were used to adjust for demographic and clinical characteristics and estimate unwarranted variation.

Setting

Observational study of claims to mandatory health insurance in Switzerland in 2014.

Participants

Enrolees eligible for the 24 health care services.

Primary outcome measures

The variances of regional random effects, also expressed as median odds ratio (MOR). Services grouped by strength and direction of recommendations were compared with Welch's t-test.

Results

The sizes of the eligible populations ranged from 1992 to 409 960 patients. MOR ranged between 1.13 for aspirin in secondary prevention of myocardial infarction to 1.68 for minor surgical procedures performed in inpatient instead of outpatient settings. Services with weak recommendations had a negligibly higher variance and MOR (difference in means [95CI%] 0.03 [-0.06, 0.11] and 0.05 [-0.11, 0.21]) compared to strong recommendations. Services with negative recommendations had a slightly higher variance and MOR (difference in means [95CI%] 0.07 [-0.03, 0.18] and 0.14 [-0.06, 0.34]) compared to positive recommendations.

Conclusions

In this exploratory study, the geographic variation in the utilization of services associated with strong versus weak and negative versus positive recommendations was not substantially different, although the difference was somewhat larger for negative versus positive recommendations. The relationships between the strength or

For peer review only - http://bmjopen.bmj.com/site/about/guidelines.xhtml

direction of recommendations and the variation may be indirect or modified by other characteristics of services. As initiatives discouraging low-value care are gaining attention worldwide, these findings may inform future research in this area.

Strengths and limitations of this study

- Although the strength and direction of recommendations is generally expected to influence the variation in clinical decisions, this is the first study to analyse this relationship quantitatively.
- The effect of the strength and direction of a recommendation on the geographic variation in health care utilization was assessed within a comprehensive set of 24 health care services.
- Unwarranted variation of the services utilization was extracted with a single standard approach.
- Indirect relationship and modifiers of the effect could not be studied.

Keywords: geographic variation in health care; unwarranted variation; clinical recommendations; clinical practice guidelines; evidence-based medicine; low-value care.

For peer review only - http://bmjopen.bmj.com/site/about/guidelines.xhtml

Background

According to the evidence-based medicine (EBM) framework, clinical decisions should be guided by research evidence, clinical circumstances, and patient preferences, and be integrated with clinical expertise [1]. If evidence is weak or lacking, patient preferences and clinical expertise have a particularly strong role in the decision [2, 3]. In a clinical practice guideline, such a situation would be reflected by a weak recommendation [2]. As patient preferences tend to vary geographically [4], and physician practice styles are also significantly influenced by the region of practice [5, 6], clinical decisions associated with less conclusive research evidence or weak recommendations may have higher geographic variation.

Surprisingly, there is little direct evidence whether weak recommendations are, in fact, associated with higher variation. The few available studies focused on a single specialty and did not quantify the variation in a uniform way, complicating the comparison of results [7, 8]. Therefore, despite many studies highlighting the substantial geographic variation in the utilization of various health care services [9–11], it is not clear, what role the components of the EBM framework play for this variation.

A second potential contributor to different services having different degrees of geographic variation is the direction of recommendation: positive (prescriptive) or negative (proscriptive), as in Choosing Wisely recommendations [12]. Negative recommendations usually concern long-used low-value practices and are based on the lack of supporting evidence or evidence of harms [12–14]. In contrast to positive recommendations, which often introduce new services or indications, negative recommendations usually challenge existing practices that are justified primarily by clinical expertise and judgement, expected to vary regionally [4]. Positive and negative recommendations have different perceived barriers to their implementation [15], which could contribute to different variation patterns as well. However, no study has directly compared the geographic variation associated with positive and negative recommendations.

The primary aim of this study was to assess whether health care services with weak recommendations are associated with higher geographic variation in utilization. In addition, a secondary aim was to test the association of geographic variation with the direction of the recommendation.

For peer review only - http://bmjopen.bmj.com/site/about/guidelines.xhtml

Methods

Study hypotheses

Although this is primarily an explorative study, we formulated two specific hypotheses. The primary hypothesis of the study was that health care services with weaker evidence, as reflected in weak recommendations in clinical guidelines, would have higher geographic variation in utilization than those with strong recommendations. The secondary hypothesis was that services with negative (proscriptive) recommendations would have higher geographic variation compared to those with positive (prescriptive) recommendations.

Selection of studied health care services

This study was part of a project assessing the geographic variation of the utilization of a set of health care services in Switzerland [16]. Studied health care services were translated from selected recommendation statements in clinical practice guidelines, following a systematic approach. We collected clinical practice guidelines of Swiss, European, and applicable international medical societies, used in Switzerland and guiding the care for major non-communicable diseases (as defined by the Swiss Federal Office of Public Health, FOPH [17]). Recommendation statements from selected clinical practice guidelines were considered pragmatically by the authors according to their clinical relevance, the expected frequency of service use, and the size of the eligible population. Identified recommended or discouraged services were then screened for feasibility of measuring the utilization in eligible populations with Swiss health insurance claims data, based on an approach described earlier [18].

We aimed for the selected services to reflect both strong and weak, positive and negative recommendations, as well as different health care services types. We focused particularly on outpatient primary health care services, as they are relevant to the biggest part of the population. However, we also included some discouraged services outside primary health care to extend the spectrum of populations investigated.

The final selection comprised 24 services, including services for screening (N=4), diagnosis (N=6), primary prevention (N=1), treatment (N=4), and secondary prevention (N=9). Definitions of the selected services are provided in Supplementary file 1.

For peer review only - http://bmjopen.bmj.com/site/about/guidelines.xhtml

Assessment of recommendations: strength and direction

Once the services were selected, their associated recommendations were formally assessed.

For each service, we selected the guideline in which the service was originally identified, and also looked up corresponding guidelines by the relevant European, American, and international clinical societies. From this set of guidelines, we selected for assessment the one that was the most applicable to Switzerland in 2014 (see Supplementary file 2 for the prioritization algorithm). We did not consider the guidelines of Swiss medical societies, as their quality of reporting is partially low [19], and they tend to be consistent with European and international guidelines. If the service was initially selected based on a guideline published after 2014, and no applicable guideline could be identified for 2014, the recommendation was automatically considered weak.

Thus, a single recommendation statement was assessed for each service. The assessment was done in duplicate by two authors (AU and HD, both medical doctors). Discordant judgements were resolved with mutual agreement in a discussion. Each recommendation was classified as strong or weak (corresponding to GRADE definition [20]), and positive or negative. The algorithm and criteria for the classification are detailed in Supplementary file 2 for the strength, and in Supplementary file 3 for the direction of a recommendation. The list of guidelines containing the recommendation statements that were assessed is provided in Supplementary file 4.

Swiss health insurance claims data

The utilization of the selected health care services was evaluated using mandatory health insurance claims data from the Helsana Group, covering approximately 1.2 million people (15% of the Swiss population). Helsana Group is one of several private companies providing mandatory health insurance in Switzerland. Eligible patient populations were identified from the patients enrolled with Helsana in 2014. Patients with incomplete address information, living in nursing homes and receiving reimbursement via lump-sums (masking some outpatient services), asylum seekers, those living outside Switzerland, and Helsana employees were excluded.

The data provided by Helsana were anonymized. According to the national ethical and legal regulations, ethical approval was not needed for this type of analysis. This was confirmed by a waiver of the competent ethics committee (Kantonale Ethikkommission Zürich, dated January 11, 2017, BASEC-Nr. Req-2017-00011).

For peer review only - http://bmjopen.bmj.com/site/about/guidelines.xhtml

Models of geographic variation

The utilization of each health care service was determined for each member of the eligible population (see Supplementary file 1 for definitions of the populations and services). For each service, the resulting binary outcome variable was modelled with a multilevel logistic regression technique, using 106 Swiss MobSpat regions ("mobilité spatiale"), as defined by the Swiss Federal Statistical Office [21], as the higher level. MobSpat regions are constructed by combining several neighbouring municipalities based on geographic and population mobility criteria, and are often used as intermediate-size units of analysis for scientific and regional policy purposes. Each study participant's residence was assigned to the corresponding MobSpat region.

Fixed effects were estimated for the following explanatory variables: age, sex, number of comorbidities (0, 1, 2, and 3 or more), and clinical characteristics of relevance for specific indicators (see Supplementary file 1). These variables are often viewed as associated with *warranted* variation [22]. In contrast, we did not adjust for variables associated with *unwarranted* variation (e.g., insurance characteristics or provider density). From each multilevel model, we extracted the variance of the regional random effects, reflecting the potentially unwarranted geographic variation. We also converted the variance to median odds ratios (MORs) for more convenient interpretation [23, 24] and plotting. MOR is interpreted as the median odds of service utilization by two individuals with identical characteristics in two randomly selected regions. As MOR is directly extrapolated from the variance, the ranking of these two parameters coincides.

Statistical analysis of the hypotheses

Variances of the regional random effects of services utilization from the models were used as data points in the final analysis of the hypotheses. Variances of services associated with weak and strong recommendations, as well as negative and positive recommendations were compared with Welch's unequal variances t-test. Mean differences and 95% confidence intervals were presented. The same analysis was also performed for MOR, to improve interpretability of detected group differences.

Although the number of the services analysed was rather small (24), the distribution of the analysed variances was deemed sufficiently close to normal to warrant the use of parametric tests. To account for the

For peer review only - http://bmjopen.bmj.com/site/about/guidelines.xhtml

small and unequal sample sizes, we used Welch's t-test, which is considered more robust in this setting [25]. Confidence intervals were not adjusted for multiple testing.

Statistical analyses were performed using R 3.6.0 [26] and MLwiN 3.01 [27] integrated in STATA 14.2 using the runmlwin package [28].

Patient and public involvement

This study was performed as part of the National Research Programme 74 "Smarter Health Care" of the Swiss National Science Foundations. Patients and public, including policy makers and healthcare services providers, are involved in interpreting, disseminating and translating the overall results of studies conducted under this programme. Representatives of patients, health care providers, health insurers, and health care policy makers are members of the advisory board of the project. They provided feedback on the planned study design and its preliminary results. Individual patients were not directly involved in the planning and conducting of this study.

Results

Characteristics of the eligible populations and the geographic variation of the services are shown in Table 1. Across the services, the sizes of the eligible populations ranged from 1 992 patients with a new disease-modifying antirheumatic drug (DMARD) prescription to 409 960 patients with recommended influenza vaccination. MOR, reflecting potentially unwarranted geographic variation in utilization of the services, ranged from 1.13 [1.02-1.29] for aspirin in secondary prevention of myocardial infarction (MI) to 1.68 [1.53-1.87] for minor surgical procedures performed in inpatient instead of outpatient settings.

For peer review only

Table 1 Characteristics of the recommended or discouraged health care services studied

Category	Health care service (abbreviated)	Utilization in eligible population	Eligible population			Recommendation		Random effects in multilevel model	
			Total N	Mean age (sd)	Female N (%)	Strength	Direction	Variance	Median odds ratio (MOR)
Screening	Colon cancer screening	5.9%	276387	58.6 (5.8)	142675 (51.6%)	Strong	Positive	0.04[0.03-0.06]	1.21[1.17-1.26]
	Breast cancer screening	20.9%	178145	61.0 (7.2)	178145 (100%)	Weak	Positive	0.22[0.16-0.29]	1.56[1.47-1.67]
	Prostate cancer screening	28.4%	145874	59.1 (6.2)	0 (0%)	Weak	Positive	0.07[0.05-0.10]	1.29[1.25-1.35]
	Osteoporosis screening	4.9%	60812	72.6 (8.7)	60812 (100%)	Weak	Positive	0.08[0.04-0.13]	1.31[1.22-1.41]
Diagnosis	DM: HbA1c test	69.6%	49198	66.6 (13.0)	22138 (45.0%)	Strong	Positive	0.17[0.12-0.23]	1.48[1.40-1.58]
	DM: renal function test	44.3%	49198	66.6 (13.0)	22138 (45.0%)	Strong	Positive	0.06[0.04-0.09]	1.27[1.22-1.33]
	DM: LDL test	44.3%	33975	60.1 (11.2)	13977 (41.2%)	Strong	Positive	0.13[0.09-0.19]	1.42[1.34-1.51]
	DM: eye examination	55.5%	49198	66.6 (13.0)	22138 (45.0%)	Weak	Positive	0.07[0.05-0.10]	1.29[1.24-1.35]
	**TSH screening**	76.1%	169232	56.8 (18.5)	111847 (66.1%)	Strong	Negative	0.18[0.13-0.25]	1.50[1.42-1.61]
	**POCR**	13.0%	47215	60.3 (17.2)	27086 (57.4%)	Strong	Negative	0.18[0.13-0.26]	1.50[1.40-1.62]
Primary prevention	Influenza vaccination	20.9%	409960	64.1 (16.3)	230202 (56.2%)	Strong	Positive	0.04[0.03-0.05]	1.20[1.17-1.24]
Treatment	**Benzodiazepines**	18.6%	243951	75.0 (7.6)	141986 (58.2%)	Strong	Negative	0.14[0.10-0.18]	1.42[1.36-1.50]
	**Proton pump inhibitors**	55.5%	153523	55.7 (17.8)	93543 (60.9%)	Weak	Negative	0.02[0.02-0.03]	1.16[1.13-1.19]
	**Inpatient procedures**	61.4%	10656	50.5 (13.7)	7719 (72.4%)	Weak	Negative	0.30[0.20-0.43]	1.68[1.53-1.87]
	**Caesarean section**	28.5%	9449	31.9 (5.1)	9449 (100%)	Weak	Negative	0.05[0.02-0.09]	1.24[1.16-1.34]
Secondary prevention	AMI: aspirin	47.0%	2232	72.4 (13.7)	801 (35.9%)	Strong	Positive	0.02[0.00-0.07]	1.13[1.02-1.29]
	AMI: statin	34.2%	2232	72.4 (13.7)	801 (35.9%)	Strong	Positive	0.14[0.06-0.27]	1.43[1.25-1.63]
	AMI: beta-blocker	42.1%	2232	72.4 (13.7)	801 (35.9%)	Strong	Positive	0.05[0.00-0.13]	1.25[1.05-1.40]
	AMI: ACE/ARB	43.8%	2232	72.4 (13.7)	801 (35.9%)	Strong	Positive	0.04[0.00-0.12]	1.21[1.03-1.39]
	AMI: P2Y12 inhibitors	46.8%	2232	72.4 (13.7)	801 (35.9%)	Strong	Positive	0.03[0.00-0.10]	1.18[1.04-1.36]
	PPI with NSAID	43.5%	95072	61.0 (16.2)	60804 (64.0%)	Strong	Positive	0.02[0.01-0.03]	1.15[1.12-1.18]
	PAD: statin	28.5%	23868	63.6 (16.5)	12113 (50.7%)	Strong	Positive	0.04[0.03-0.07]	1.22[1.17-1.28]
	Afib: anticoagulation	27.5%	8291	80.8 (7.9)	4037 (48.7%)	Strong	Positive	0.05[0.02-0.09]	1.24[1.16-1.33]
	GCC with new DMARD	58.7%	1992	59.2 (15.3)	1369 (68.7%)	Weak	Positive	0.06[0.01-0.18]	1.27[1.07-1.49]

Health care services, highlighted in bold, are associated with a negative recommendation. Utilization was assessed within one year, 2014, including for services that are recommended less frequently (e.g., colon cancer screening).

For peer review only

For peer review only - http://bmjopen.bmj.com/site/about/guidelines.xhtml

SD – standard deviation, N – number, DM – diabetes mellitus, LDL – low density lipid, TSH – thyroid-stimulating hormone, POCR – preoperative chest radiography, AMI – acute myocardial infarction, ACE/ARB – angiotensin-converting enzyme inhibitors or angiotensin II receptor blockers, PPI – proton pump inhibitors, NSAID – nonsteroidal anti-inflammatory drugs, PAD – peripheral artery disease, Afib – atrial fibrillation, GCC – glucocorticosteroid drugs, DMARD – disease-modifying antirheumatic drug.

For peer review only

For three services, a major guideline relevant in 2014 in Switzerland could not be identified (long-term use of proton pump inhibitors, minor inpatient surgery procedures, elective Caesarean section). A total of eight services had weak, and six services had negative underlying recommendations. Median MOR was 1.29 for services with weak and 1.25 for services with strong recommendations; 1.26 for services with positive and 1.46 for services with negative recommendations (Figure 1).

Figure 1 Geographic variation of the health care services grouped by strength (A) and direction (B) of recommendations

A Weak and strong recommendations; B Positive and negative recommendations. MOR – median odds ratio. Boxplots depict the interquartile range of values (upper and lower hinges), and the median value.

Based on Welch's t-test, the difference in mean variances [95CI%] of services with weak and strong recommendations was 0.03 [-0.06, 0.11], and the difference in mean MOR was 0.05 [-0.11, 0.21]. The difference in mean variances [95CI%] of services with negative and positive recommendations was 0.07 [-0.03, 0.18], and the difference in mean MOR was 0.14 [-0.06, 0.34].

Discussion

We did not find a direct association between the strength of clinical recommendation and the geographic variation in the utilization of 24 health care services. The geographic variation in the utilization of services with underlying negative recommendations was slightly higher than for those with positive recommendations, and for services with underlying weak recommendations than for those with strong recommendations. The difference was larger for negative vs positive recommendations; however, both differences were not statistically significant. In general, moderate potentially unwarranted geographic variation was observed, with MOR smaller than 1.50 for all but one service.

At least two other studies have to some extent examined the association between the strength of recommendations and the variation in adherence, each focusing on a single clinical specialty. In et al [7], examining a set of recommendations in oncology, found higher variation in the utilization of services associated

For peer review only - http://bmjopen.bmj.com/site/about/guidelines.xhtml

with a lower level of evidence. However, this study focused not on regional but on inter-institutional variation, comparing two groups of providers. In contrast to this study and in agreement with our results, Mayer et al [8] found that surgeon practices in knee and hip arthroplasty in Australia varied regardless of the strength of evidence available. Potentially, different clinical areas could be associated with different barriers to guideline implementation, modifying the relationship between recommendations and variation.

To better understand why a direct association of recommendation strength and variation in adherence was not observed, it may be useful to revisit the EBM framework [1]. The EBM framework is *normative* and defines how clinical decisions *should* be made [1, 29]. However, this may not always coincide with how decisions *are* made – a process analysed by *descriptive* theories [29]. In fact, the EBM model has been developed as a conceptual rather than practical guidance of evidence implementation [1], and has not yet generated a coherent theory of clinical decision making, and in particular, of how evidence is incorporated [30]. Thus, although a direct relationship between the strength of recommendation and the geographic variation of service utilization would be encouraged by the normative EBM framework, it may not always be observed.

There are numerous reasons why even strong recommendations [31, 32], or conclusive research evidence more broadly [33], may not directly translate into clinical practice. Research on knowledge translation has identified multiple barriers at different levels of the health care system, including structural, organizational, peer-group, and professional factors [34] – many of which depend on the specific context where a service is provided, and thus may vary geographically. Knowledge transfer processes are highly non-linear and rely on triggering mechanisms [35]. Factors external to research evidence significantly affect translation – potentially creating large geographic heterogeneity even within services with strong recommendations. Finally, strong recommendations sometimes describe care with varying patient preferences. For example, although colon cancer screening is strongly recommended, patient preferences for test attributes and modalities vary significantly [36, 37].

An influential framework, explaining different degrees of variation between health care services, has been proposed by Wennberg and colleagues [38]. According to this framework, services are classified into effective, preference-sensitive, and supply-sensitive care. Effective care (services based on solid evidence, so that virtually all patients would choose them) largely corresponds to services with strong recommendations, as

For peer review only

defined by GRADE and applied in this study [2]. Preference-sensitive care partly corresponds to services with weak recommendations, as they both imply trade-offs of risks and benefits of multiple options of care [38]. The utilization of preference-sensitive surgical procedures usually has higher variation than of those associated with effective care [39]. In contrast, supply-sensitive care defines the frequency, setting, and intensity of care provision rather than specific types of health care services. It is associated with high, supply-related variation, but is rarely discussed in guidelines [39], and therefore, could not be included in our study. However, the service of minor surgeries performed as inpatient instead of outpatient procedures could be considered close to the supply-sensitive category. In fact, it had the highest MOR (1.68) in our study.

Regarding the secondary hypothesis, we found that services associated with negative recommendations had slightly higher geographic variation. We did not find other studies directly comparing the regional variation of services with the direction of recommendations. Few studies, focusing mostly on low-value care, have reported MOR as an expression of geographic variation, further limiting the comparison. For example, in a study by Badgery-Parker et al [40], services discouraged by *Choosing Wisely* were shown to have regional MOR from 1.1 to 2.6 – a range that includes all of our observed MORs.

Negative recommendations usually address a widespread service that lacks supporting evidence of benefit or the benefit is outweighed by harms [2]. In contrast to services with positive recommendations, which are introduced after supporting evidence is produced, services with negative recommendations typically become part of the clinical practice *before* evidence is sufficient to rule out their overall benefit. Therefore, their use could be related more to clinical expertise and practice, and could be expected to vary locally. Indeed, the barriers to implementing positive and negative recommendations seem to be different [15] – signalling that the pathways how they are interpreted and integrated into clinical decisions might also be different. As Choosing Wisely and similar initiatives are increasingly gaining attention [41], our finding of higher geographic variation associated with negative recommendations may inform future research and implementation strategies.

This exploratory study has several limitations. First, although we aimed at a balanced selection of clinical fields and service types, the number (24) and range of studied services was limited by the data source, leading to somewhat unbalanced groups of strong and weak, positive and negative recommendations. Swiss claims data lack information on outpatient diagnoses, inpatient treatment details, and clinical information such as test

For peer review only - http://bmjopen.bmj.com/site/about/guidelines.xhtml

results [18]. Lack of clinical information also meant that some populations were not as specific as defined by the recommendation. For example, beta-blockers and angiotensin-converting-enzyme (ACE) inhibitors are recommended after a myocardial infarction contingent on heart failure and left ventricular dysfunction. As such clinical details were unavailable, we had to rely on them being present in the majority of the hospitalized myocardial infarction cases and distributed equally geographically. However, we believe that estimates of variation are accurate, as each of the 24 data points was generated by multilevel modelling of utilization in populations of 85000 patients on average, including all major explanatory variables such as age, sex, and indicators of morbidity. Second, the services studied were unavoidably different by characteristics other than the strength or direction of recommendation, such as service type or clinical area, potentially resulting in confounding. Indeed, although distributed among all recommendation types, diagnostic services had somewhat higher regional variation in utilization compared to treatment services (see Supplementary file 5). Although most of the selected services are delivered by primary care providers, their varied nature also means that the applied MobSpat regional units might not capture the regional variation equally well. Third, both the observed utilization and its geographic variation depend on the definition of the service and population [42]. We aimed to measure the *unwarranted* variation in utilization by using service-specific denominators (eligible populations) and adjusting for relevant clinical characteristics. How exactly *unwarranted* and *warranted* variation should be defined and measured, and what adjustments are necessary to differentiate them, is debated [22, 43]. Fourth, the grouping of recommendations by strength and direction was partly subjective, although we tried to make it reproducible with a clear algorithm, implemented in duplicate. Unfortunately, many different systems for evaluating the strength of recommendations exist [44], which cannot be easily reconciled, and the most prominent, GRADE approach, is not always explicitly used.

To explore the studied questions further, the sample of services could be expanded to inpatient and specialist care. Further, a meta-study of the numerous individual studies of geographic variation in health care services could be undertaken. However, this would currently be challenging, as studies choose different adjustment variables and specificity of studied populations, and report the variation in different quantitative forms (e.g., MOR, systematic component of variation, range). Furthermore, there is a need for qualitative studies of the reasons for the variability of clinical decisions, and how clinical expertise in these decisions

For peer review only - http://bmjopen.bmj.com/site/about/guidelines.xhtml

interacts with evidence, clinical circumstances, and patient preferences. Qualitative evidence could help to generate more complex hypotheses for further quantitative studies, built on a finer understanding of all the factors influencing the variability of clinical decisions. Specialty-specific sets of services could also be further investigated.

Conclusions

In this exploratory study of 24 health care services mostly in the outpatient primary care setting, we did not observe a significant difference in the degree of geographic variation in utilization of services associated with strong versus weak recommendations. Services associated with negative recommendations had slightly, although also not statistically significantly, higher geographic variation. The relationship between the strength of recommendations and the variation may be indirect or significantly modified by other characteristics of services, such as service type or clinical area. As initiatives discouraging low-value care are gaining attention worldwide, these findings may inform future research in this area.

Funding

This work was supported by the Swiss National Science Foundation (SNSF) National Research Program "Smarter Health Care" (NRP 74), as part of project number 26, grant number 407440_167349.

Competing interests

MS declares a grant from Helsana Insurance Group, outside the submitted work. Helsana Group provided support in the form of salaries for authors BB, EB and CB, but did not have any additional role in the study design, data collection and analysis, decision to publish, or preparation of the manuscript. The other authors declare no competing interests.

Authors' contributions

MS, VvW and HD developed the underlying study program. AU and HD developed study design, with support from all authors. AU, WW, CB, EB, BB did data extraction, preparation and management. WW, MS and OG

For peer review only

developed multilevel models applied in this study. AU and WW analysed the data. AU drafted the manuscript, with major inputs from HD and MS, and contributions from all authors. All authors read and approved the final manuscript.

Data sharing statement

The data underlying this study cannot be shared publicly because they are the property of Helsana (https://www.helsana.ch/en/helsana-group), and have restricted public access on grounds of patient privacy. The data are managed by Helsana and subsets of the database are available for researchers after request and under specific conditions. Data are available from Helsana (gesundheitskompetenz@helsana.ch) for researchers who meet the criteria for access to confidential data. Helsana will consider the possibilities of the research proposal and decide to grant access if the research questions can be answered with use of the Helsana data. When requests are granted, data are accessible only in a secure environment.

References

1. Haynes RB, Devereaux PJ, Guyatt GH. Clinical expertise in the era of evidence-based medicine and patient choice. Evid Based Med. 2002;7:36–8.

2. Andrews J, Guyatt G, Oxman AD, Alderson P, Dahm P, Falck-Ytter Y, et al. GRADE guidelines: 14. Going from evidence to recommendations: The significance and presentation of recommendations. J Clin Epidemiol. 2013;66:719–25. doi:10.1016/j.jclinepi.2012.03.013.

3. Andrews JC, Schünemann HJ, Oxman AD, Pottie K, Meerpohl JJ, Coello PA, et al. GRADE guidelines: 15. Going from evidence to recommendation - Determinants of a recommendation’s direction and strength. J Clin Epidemiol. 2013;66:726–35. doi:10.1016/j.jclinepi.2013.02.003.

4. Cutler D, Skinner JS, Stern AD, Wennberg D. Physician beliefs and patient preferences: A new look at regional variation in health care spending. Am Econ J Econ Policy. 2019;11:192–221.

5. Finkelstein A, Gentzkow M, Williams H. Sources of Geographic Variation in Health Care: Evidence From PatientMigration. Q J Econ. 2016;131:1681–726.

6. Molitor D. The evolution of physician practice styles: Evidence from cardiologist migration. Am Econ J Econ

For peer review only

Policy. 2018;10:326–56.

7. In H, Neville BA, Lipsitz SR, Corso KA, Weeks JC, Greenberg CC. The role of National Cancer Institute-designated cancer center status: observed variation in surgical care depends on the level of evidence. Ann Surg. 2012;255:890–5. doi:10.1097/SLA.0b013e31824deae6.

8. Mayer M, Naylor J, Harris I, Badge H, Adie S, Mills K, et al. Evidence base and practice variation in acute care processes for knee and hip arthroplasty surgeries. PLoS One. 2017;12:e0180090. doi:10.1371/journal.pone.0180090.

9. Corallo AN, Croxford R, Goodman DC, Bryan EL, Srivastava D, Stukel TA. A systematic review of medical practice variation in OECD countries. Health Policy (New York). 2014;114:5–14. doi:10.1016/j.healthpol.2013.08.002.

10. The Dartmouth Atlas of Health Care. https://www.dartmouthatlas.org/. Accessed 11 May 2020.

11. Public Health England. Atlas of Variation. https://fingertips.phe.org.uk/profile/atlas-of-variation. Accessed 11 May 2020.

12. Cassel CK, Guest JA. Choosing wisely: Helping physicians and patients make smart decisions about their care. JAMA - Journal of the American Medical Association. 2012;307:1801–2.

13. Elshaug AG, Watt AM, Mundy L, Willis CD. Over 150 potentially low-value health care practices: an Australian study. Med J Aust. 2012;197:556–60.

14. Prasad V, Vandross A, Toomey C, Cheung M, Rho J, Quinn S, et al. A decade of reversal: An analysis of 146 contradicted medical practices. Mayo Clin Proc. 2013;88:790–8.

15. Carlsen B, Glenton C, Pope C. Thou shalt versus thou shalt not: A meta-synthesis of GPs' attitudes to clinical practice guidelines. Br J Gen Pract. 2007;57:971–8.

16. National Research Programme 74. Project 26: How do guidelines and recommendations influence medical treatment? http://www.nfp74.ch/en/projects/healthcare-across-sectors/project-schwenkglenks. Accessed 15 Apr 2020.

17. Swiss Federal Office of Public Health and Swiss Conference of Cantonal Health Directors. Nationale Strategie Prävention nichtübertragbarer Krankheiten (NCD-Strategie) 2017-2024 [National Strategy of Non-communicable Diseases]. 2016.

For peer review only

For peer review only - http://bmjopen.bmj.com/site/about/guidelines.xhtml

18. Ulyte A, Bähler C, Schwenkglenks M, von Wyl V, Gruebner O, Wei W, et al. Measuring diabetes guideline adherence with claims data: systematic construction of indicators and related challenges. BMJ Open. 2019;9:e027138. doi:10.1136/bmjopen-2018-027138.

19. Bachmann L, Ulytė A, Dressel H. Clinical practice guidelines of medical societies in Switzerland: analysis of the current state. Swiss Med Wkly. 2019;149:w20134.

20. Guyatt GH, Oxman AD, Kunz R, Falck-Ytter Y, Vist GE, Liberati A, et al. GRADE: Going from Evidence to Recommendations. BMJ. 2008;336:1048–51.

21. Bundesamt für Statistik. MS-Regionen. https://www.bfs.admin.ch/bfs/de/home/statistiken/raum-umwelt/nomenklaturen/msreg.assetdetail.415729.html. Accessed 11 May 2020.

22. Sutherland K, Levesque J. Unwarranted clinical variation in health care: Definitions and proposal of an analytic framework. J Eval Clin Pract. 2019;:jep.13181. doi:10.1111/jep.13181.

23. Larsen K, Petersen JH, Budtz-Jørgensen E, Endahl L. Interpreting parameters in the logistic regression model with random effects. Biometrics. 2000;56:909–14.

24. Larsen K, Merlo J. Appropriate Assessment of Neighborhood Effects on Individual Health: Integrating Random and Fixed Effects in Multilevel Logistic Regression. Am J Epidemiol. 2005;161:81–8.

25. Ruxton GD. The unequal variance t-test is an underused alternative to Student's t-test and the Mann-Whitney U test. Behav Ecol. 2006;17:688–90.

26. R Core Team. R: A language and environment for statistical computing. R Foundation for Statistical Computing, Vienna, Austria. 2020. https://www.r-project.org/. Accessed 17 Dec 2020.

27. Charlton, C, Rasbash, J, Browne, WJ, Healy, M, Cameron B. MLwiN Version 3.01. Centre for Multilevel Modelling, University of Bristol. 2017.

28. Leckie G, Charlton C. Runmlwin: A program to run the MLwiN multilevel modeling software from within Stata. J Stat Softw. 2013;52:1–40.

29. Djulbegovic B, Elqayam S, Dale W. Rational decision making in medicine: Implications for overuse and underuse. J Eval Clin Pract. 2018;24:655–65. doi:10.1111/jep.12851.

30. Djulbegovic B, Guyatt GH. Progress in evidence-based medicine: a quarter century on. The Lancet. 2017;390:415–23.

For peer review only

For peer review only - http://bmjopen.bmj.com/site/about/guidelines.xhtml

419 31. Francke AL, Smit MC, De Veer AJE, Mistiaen P. Factors influencing the implementation of clinical guidelines for health care professionals: A systematic meta-review. BMC Med Inform Decis Mak. 2008;8:38. doi:10.1186/1472-6947-8-38.

422 32. Lugtenberg M, Zegers-Van Schaick JM, Westert GP, Burgers JS. Why don't physicians adhere to guideline recommendations in practice? An analysis of barriers among Dutch general practitioners. Implement Sci. 2009;4:1–9.

425 33. Wallace J, Nwosu B, Clarke M. Barriers to the uptake of evidence from systematic reviews and meta-analyses: A systematic review of decision makers' perceptions. BMJ Open. 2012;2:e001220.

427 34. Grimshaw JM, Eccles MP, Lavis JN, Hill SJ, Squires JE. Knowledge translation of research findings. Implement Sci. 2012;7:50. doi:10.1186/1748-5908-7-50.

429 35. Braithwaite J, Churruca K, Long JC, Ellis LA, Herkes J. When complexity science meets implementation science: A theoretical and empirical analysis of systems change. BMC Med. 2018;16:63. doi:10.1186/s12916-018-1057-z.

432 36. Marshall DA, Johnson FR, Phillips KA, Marshall JK, Thabane L, Kulin NA. Measuring Patient Preferences for Colorectal Cancer Screening Using a Choice-Format Survey. Value Heal. 2007;10:415–30.

434 37. Mansfield C, Tangka FKL, Ekwueme DU, Smith JL, Guy GP, Li C, et al. Stated Preference for Cancer Screening: A Systematic Review of the Literature, 1990-2013. Prev Chronic Dis. 2016;13:E27. doi:10.5888/pcd13.150433.

436 38. Wennberg JE, Fisher ES, Skinner JS. Geography and the debate over medicare reform. Health Affairs. 2003;22 SUPPL.

438 39. Wennberg JE. Tracking medicine : a researcher's quest to understand health care. Oxford University Press; 2010.

440 40. Badgery-Parker T, Feng Y, Pearson S-AA, Levesque J-FF, Dunn S, Elshaug AG. Exploring variation in low-value care: a multilevel modelling study. BMC Health Serv Res. 2019;19:345. doi:10.1186/s12913-019-4159-1.

442 41. Levinson W, Kallewaard M, Bhatia RS, Wolfson D, Shortt S, Kerr E a. "Choosing Wisely": a growing international campaign. BMJ Qual Saf. 2015;24:167–74. doi:10.1136/bmjqs-2014-003821.

444 42. Schwartz AL, Landon BE, Elshaug AG, Chernew ME, McWilliams JM. Measuring low-value care in Medicare. JAMA Intern Med. 2014;174:1067–76. doi:10.1001/jamainternmed.2014.1541.

For peer review only - http://bmjopen.bmj.com/site/about/guidelines.xhtml

43. Harrison R, Manias E, Mears S, Heslop D, Hinchcliff R, Hay L. Addressing unwarranted clinical variation: A rapid review of current evidence. J Eval Clin Pract. 2019;25:53–65. doi:10.1111/jep.12930.

44. Atkins D, Eccles M, Flottorp S, Guyatt GH, Henry D, Hill S, et al. Systems for grading the quality of evidence and the strength of recommendations I: Critical appraisal of existing approaches. BMC Health Serv Res. 2004;4:1–7.

Supplementary files

Supplementary file 1 Definitions and descriptions of the studied health care services and eligible populations

Supplementary file 2 Algorithm and criteria for the assessment of the strength of recommendation

Supplementary file 3 Algorithm and criteria for the assessment of the direction of recommendation

Supplementary file 4 List of guidelines selected for the study, describing the services analysed

Supplementary file 5 Regional variation of diagnostic and treatment services studied

For peer review only

For peer review only - http://bmjopen.bmj.com/site/about/guidelines.xhtml

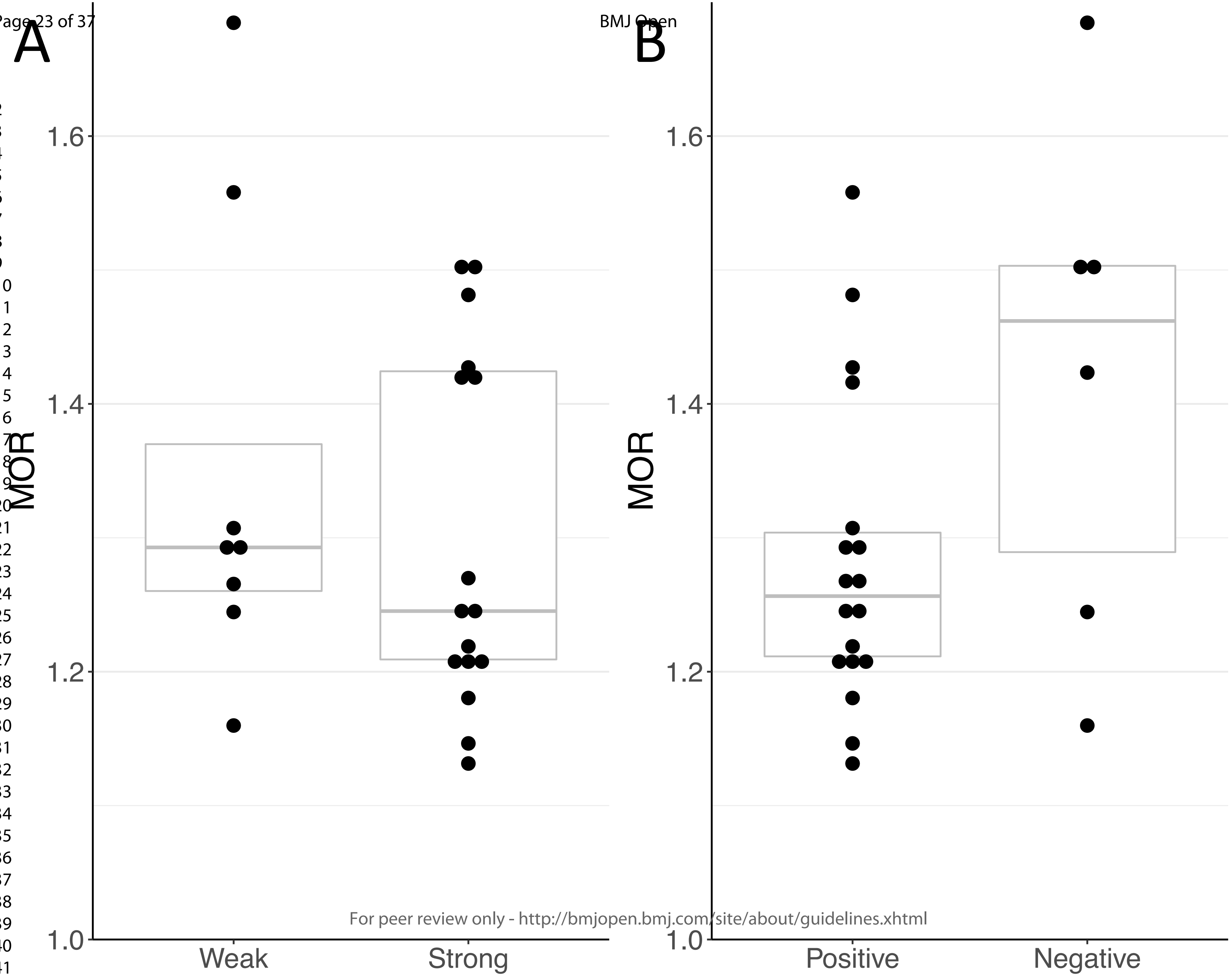
A
B
MOR
MOR
1.6
1.4
1.2
1.0
Weak
Strong
Positive
Negative

Additional file 1 Definitions and descriptions of the studied health care services and eligible populations

Category	Health care service	Service description and frequency	Eligible population	Recommendation for the health care service	Specific clinical explanatory variables	Clinical codes used for identification of the health care service
Screening	Colon cancer screening	Colonoscopy/ year	Anyone 50-69 years old	Colonoscopy should be done every 10 years for people 50-69 years old.	Previous treatment of cancer or inflammatory bowel disease, hospitalization with colon disease in the last year	Colonoscopy: 19.06 (TM Kapitel); G48% (DRG); 45.23, 45.25, 48.29.1%, 48.29.2% (CHOP)
	Breast cancer screening	Mammography/ year	50-74 years old women	Mammography should be done every 2 years for 50-74 years old women.	Previous treatment of breast or other cancer	Mamography: 39.1310, 39.1320, 39.1307, 39.1308, 39.1300, 39.1305, 39.1306 (TM); TZ
	Prostate cancer screening	Prostate-specific antigen (PSA) testing/ year	50-70 years old men	Early detection of prostate cancer (opportunistic screening) should be offered to the well-informed man.	Previous treatment of cancer, hospitalization with prostate disease in the last year	PSA testing: 1626.00 (Ana)
	Osteoporosis screening	Dual-energy x-ray absorptiometry (DXA)/ year	Women over 60 and with risk factors[a] of spontaneous fractures	DXA densitometry is recommended for postmenopausal women with spontaneous fractures or increased risk of them.	Presence of more than one risk factor	DXA densitometry: 39.1950, 39.2140, 39.2150, 39.2160 (TM)

For peer review only

Diagnosis	DM: HbA1c test	Glycated haemoglobin (HbA1c) test twice/ year	>18-year-old drug-treated diabetes patients	HbA1c test should be done for diabetes patients at least twice a year.	Oral diabetes medication or insulin	HbA1c test: 1363.00, 1363.01 (Ana)
	DM: renal function test	Albuminuria and serum creatinine tests/ year	>18-year-old drug-treated diabetes patients	Albuminuria and serum creatinine tests should be done for diabetes patients at least once a year.	Oral diabetes medication or insulin	Albuminuria: 1023.00, 1023.01, 1739.00, 1739.01, 1740.00, 1740.01 (Ana)<br>Serum creatinine: 1509.00, 1509.01 (Ana)
	DM: LDL test	Low-density lipoprotein (LDL) test/ year	19-75-year-old drug-treated diabetes patients	LDL test should be done for diabetes patients at least once a year.	Oral diabetes medication or insulin	LDL test: 1521.00 (Ana)<br>Total cholesterol test: 1230.00, 1230.01 (Ana)<br>HDL test: 1410.01, 1410.10 (Ana)<br>Triglycerides test: 1731.01, 1731.00 (Ana)
	DM: eye examination	Ophthalmologist visit/ year	>18-year-old drug-treated diabetes patients	Eye exam should be performed for diabetes patients at least once a year.	Oral diabetes medication or insulin	Outpatient visit with ophthalmologist: (sub group "Ophthalmologie" in Swiss care provider registry sasis.ch)
	TSH screening	Thyroid-stimulating hormone (TSH) test without T3 and T4 tests on the same day	>18-year-old persons without thyroid disease[b] and receiving TSH test	TSH should be measured as an initial screening test for hypo/hyperthyroidism, while T3 and T4 test should follow if TSH is abnormal.	-	TSH test: 1718.10 (Ana)<br>T3 or T4 test: 1732.00, 1720.00, 733.00, 1721.00 (Ana)

For peer review only - http://bmjopen.bmj.com/site/about/guidelines.xhtml

	POCR	Outpatient preoperative chest radiography (POCR) up to 2 months before surgery	>18-year-old patients with inpatient surgical procedures	Routine chest radiography is not recommended before surgery.	-	Chest radiography: 39.0190 (TM)
Primary prevention	Influenza vaccination	Influenza outpatient vaccination/ year	People over 65 years old or with a specified chronic condition[c]	People over 65 years old and patients with chronic conditions, specified by Federal Office of Public Health, should be vaccinated against influenza every year.	Hospitalization with pneumonia in the last year	Influenza vaccination: J07BB02 (ATC)
Treatment	Benzodiazepines	Cumulative prescription of benzodiazepines (BZD) for >8 weeks/ year	Anyone over 65 years old	Long-term use of benzodiazepines and other hypnotics is discouraged for old patients.	Treated epilepsy, stay in a nursing home in the last year, hospitalization in the last year with a diagnosis indicative of justified benzodiazepine use	Benzodiazepines and other hypnotics: N03AE01, N05BA%, N05CD%, N05BB%, N05BE%, N05CA%, N05CB%, N05CC%, N05CF%, N05CH%, N05CM%, N05CX% (ATC)
	Proton pump inhibitors	Cumulative prescription of proton pump inhibitors (PPI) or H2 histamine receptor antagonists (H2) for >8 weeks/ year	>18-year-old persons receiving PPI or H2 drugs	PPI should not be used at maximal dose for prolonged periods of time.	-	PPI or H2: A02BC%, A02BD%, M01AE52, A02BA% (ATC)
	Inpatient procedures	Specified surgical procedures[d] done in	>18-year-old patients with	If none of the special conditions apply, certain surgical	-	

For peer review only - http://bmjopen.bmj.com/site/about/guidelines.xhtml

		the outpatient setting	specified surgical procedures (either as in- or outpatient)	procedures should be done in the outpatient setting.		
	Caesarean section	Caesarean section (C-section)	>18-year-old women giving birth without absolute indications[e] for C-section	C-section should not be performed unless absolute or relative indications are present.	-	C-section: 74.0%, 74.1%, 74.2%, 74.4%, 74.99 (CHOP); O01A, O01B, O01C, O01D, O01E, O01F (DRG); 22.2120, 22.2130, 22.2410, 22.2420 (TM)
Secondary prevention	AMI: aspirin	Aspirin prescription within 2 weeks after acute myocardial infarction (AMI)	>18-year-old patients with AMI[f]	All myocardial infarction patients should take aspirin long-term.	Hospitalization for stroke or bleeding event or prescribed anticoagulation in the last year	Aspirin: B01AC06 (ATC)
	AMI: statin	High-dose statin prescription within 2 weeks after AMI	>18-year-old patients with AMI [f]	All myocardial infarction patients should get statins long-term.	Hospitalization for stroke in the last year	High-dose statins: C10AA05, C10AA07 (ATC)
	AMI: beta-blocker	Beta-blocker prescription within 2 weeks after AMI	>18-year-old patients with AMI [f]	All myocardial infarction patients with heart failure or impaired function should get beta-blockers long-term.	Hospitalization with heart failure diagnosis in the last year	Beta-blockers: C07% (ATC)
	AMI: ACE/ARB	Angiotensin-converting enzyme (ACE) or angiotensin	>18-year-old patients with AMI [f]	All myocardial infarction patients with heart failure or impaired function should get	-	ACE or ARB medication: C09% (ATC)

For peer review only - http://bmjopen.bmj.com/site/about/guidelines.xhtml

		receptor blocker (ARB) antihypertensive medication prescription within 2 weeks after AMI		ACE or ARB antihypertensive medication long-term.		
	AMI: P2Y12 inhibitors	P2Y12 antiplatelet drug[g] prescription within 2 weeks after AMI	>18-year-old patients with AMI[f]	All myocardial infarction patients should get P2Y12 antiplatelet drugs for at least 1-12 months according to the bleeding risk profile and AMI treatment.	Hospitalization for a bleeding event or prescribed anticoagulation in the last year	P2Y12 drugs: B01AC04, B01AC22, B01AC24 (ATC)
	PPI with NSAID	PPI prescription within 1 month or up to 3 months before initial long-term nonsteroidal anti-inflammatory drug (NSAID) prescription	>18-year-old patients with a cumulative NSAID prescription of >8 weeks at maximal dose	Patients taking long-term NSAID and with risk factors for gastric ulcer[h] should also take PPI.	Concurrent use of antiplatelet, anticoagulation drugs or oral glucocorticoids, hospitalization for bleeding event in the last year.	NSAID: M01A% (ATC)<br>PPI: A02BC%, A02BD%, M01AE52 (ATC)
	PAD: statin	Prescription of statins within 3 months after peripheral artery disease (PAD) identification	>18-year-old patients undergoing diagnostic or treatment	Statins are recommended for all patients with PAD.	-	Statins: C10AA%, C10B% (ATC)

For peer review only - http://bmjopen.bmj.com/site/about/guidelines.xhtml

			procedures for PAD[i]			
	Afib: anticoagulation	Oral anticoagulation prescription within 2 weeks after atrial fibrillation (Afib) identification	>18-year-old patients with atrial fibrillation diagnosis and additional risk factors[j]	All patients with atrial fibrillation should be prescribed oral anticoagulation for embolic events prevention according to the CHA_2DS_2-VASc score.	-	Oral anticoagulation: B01AE07, B01AF01, B01AF02, B01AF03, B01AA04, B01AA07 (ATC)
	GCC with new DMARD	Glucocorticoid (GCC) prescription within 1 month or up to 3 months before disease-modifying antirheumatic drug (DMARD) prescription	>18-year-old patients with a new prescription of DMARD by a rheumatologist	Short-term glucocorticoids should be taken with newly prescribed DMARD.	-	Glucocorticoids: H02% (ATC) DMARD: L01BA01, L04AX03, M01CX01, L04AA13, M01CX02, P1BA02, P01BA01, M01CC01, L01AA01, M01CB01, L04AX01 (ATC)

a. Recent distal radius, proximal humerus, vertebral or femoral fracture, use of drugs increasing the risk of osteoporosis, use of oral glucocorticoids, diabetes, ankylosing spondylitis, osteogenesis imperfecta, rheumatoid arthritis, inflammatory bowel disease, Cushing's disease, alcohol or nicotine abuse, chronic liver disease, gastrectomy, malnutrition, hypogonadism, hyper- or hypothyroidism, and hyperparathyroidism. Patients currently treated or diagnosed with osteoporosis were excluded.

b. Hyperthyroidism, hypothyroidism, goitre or thyroiditis.

c. Cardiovascular disease, chronic pulmonary disease, diabetes, chronic liver disease, renal failure, immune deficiency, systemic neurologic disorders.

d. Varicose veins ligation and stripping, surgical procedures of haemorrhoids, inguinal hernia and cervix, knee arthroscopy and meniscectomy, tonsillectomy.

e. Placental, umbilical cord or fetal pathology, HIV or genital HSV infection, or multiple pregnancy.

f. Inpatient treatment with a diagnosis of acute myocardial infarction (AMI).

g. Clopidogrel, prasugrel or ticagrelor.

For peer review only

For peer review only - http://bmjopen.bmj.com/site/about/guidelines.xhtml

h. Concurrent use of antiplatelet, anticoagulant drugs, oral glucocorticoids or recent hospitalization with any major bleeding.

i. Peripheral artery disease (PAD) or carotid stenosis diagnosed during an inpatient stay, amputation of lower or upper extremity, thrombectomy, stenting or other procedures in peripheral arteries, specialized diagnostic ultrasound, magnetic resonance tomography (MRI) angiography, computer tomography (CT) angiography or angiography of peripheral arteries.

j. Risk factors (congestive heart failure, hypertension, age 65-74 or ≥75 years old, diabetes, previous stroke, transient ischemic attack, or thromboembolism, cardiovascular disease, female sex) were extracted from available claims data and summed according to CHA2DS2-VASc score. Patients with CHA2DS2-VASc score of ≥2 for males and ≥3 for females were included.

DM – diabetes mellitus, HbA1c – Glycated haemoglobin, LDL – low density lipid, TSH – thyroid-stimulating hormone, T3 and T4 – triiodothyronine and thyroxine, POCR – preoperative chest radiography, BZD – benzodiazepines, PPI – proton pump inhibitors, H2 – H2 histamine receptor antagonists, C-section – Caesarean section, AMI – acute myocardial infarction, ACE/ARB – angiotensin-converting enzyme inhibitors or angiotensin II receptor blockers, NSAID – nonsteroidal anti-inflammatory drugs, PAD – peripheral artery disease, Afib – atrial fibrillation, GCC – glucocorticosteroid drugs, DMARD – disease-modifying antirheumatic drug.

Ana – Analysenliste, Swiss outpatient laboratory test codes; ATC - Anatomical Therapeutic Chemical Classification System, code and quantity of a prescription drug; CHOP - Schweizerische Operationsklassifikation, a classification of inpatient procedures; DRG - Swiss Diagnosis Related Groups, a classification of inpatient cases, based on diagnoses, procedures and other clinical information; ICD - International Classification of Diseases, 10th revision, German Modification, codes for primary and secondary diagnoses for each hospitalization episode of an inpatient; TM – Tarmed, Swiss classification of outpatient procedures and services; TM Kapitel – Tarmed chapter codes; TZ – Tarifziffer, further codes representing reimbursement of screening services within cantonal breast cancer screening programs.

For peer review only

Additional file 2 Algorithm and criteria for the assessment of the strength of recommendation

N of authors	Steps
Single	1. Identify the relevant medical societies for each selected health care service.<br>2. Look up the European or international medical societies' websites and journals, and identify relevant guidelines published before 2014. In addition, look up if Swiss federal legislation guidelines exist by 2014.<br>3. If none found, look up American medical society and identify relevant guidelines published before 2014.<br>4. If none found, consider the recommendation **weak**.
In duplicate	5. Once the guideline and the recommendation statement are located, classify the recommendation into strong or weak[a].<br>- **Strong recommendation** implies that the desirable effects of adherence to a recommendation outweigh the undesirable effects.<br>- That means that most informed patients would choose the recommended management and that clinicians can structure their interactions with patients accordingly.<br>- For clinicians, that would mean that most patients should receive the recommended course of action.<br>For patients, that would mean that most people in such a situation would want the recommended course of action and only a small proportion would not; patients should request discussion if the intervention is not offered.<br>- **Weak recommendation** implies that the desirable effects of adherence to a recommendation probably outweigh the undesirable effects, but the guideline panel is less confident.<br>- Thus, a weak recommendation is conditional or optional, and means that patients' choices will vary according to their values and preferences, and clinicians must ensure that patients' care is in keeping with their values and preferences.<br>- For clinicians, that would mean that they should recognize that different choices will be appropriate for different patients and that they must help each patient to arrive at a management decision consistent with her or his values and preferences.<br>For patients, that would mean that most people in such situation would want the recommended course of action, but many would not.

a adapted from: Guyatt GH, Oxman AD, Kunz R, Falck-Ytter Y, Vist GE, Liberati A, et al. GRADE: Going from Evidence to Recommendations. BMJ 2008;336:1048–51.

For peer review only - http://bmjopen.bmj.com/site/about/guidelines.xhtml

Additional file 3 Algorithm and criteria for the assessment of the direction of recommendation

N of authors	Steps	
In duplicate	1.	Once the guideline and the recommendation statement are located (see Additional file 2), classify the recommendation into positive and negative.
	-	**Positive** recommendation encourages the use of a health care service in a given population.
	-	**Negative** recommendation discourages the use of a health care service in a given population (e.g., contains negative indicatory words, such as *not, no, never*)

For peer review only

Additional file 4 List of guidelines selected for the study, describing the services analysed

Recommendation	Reference	Comment
Colon cancer screening	[1]	
Breast cancer screening	[2]	
Prostate cancer screening	[3]	
Osteoporosis screening	[4]	
DM: HbA1c test	[5]	
DM: renal function test	[5]	
DM: LDL test	[5]	
DM: eye examination	[5]	
TSH screening	[6,7]	
POCR	[8]	
Influenza vaccination	[9]	
Benzodiazepines	[10]	
Proton pump inhibitors	-	Swiss national guideline since 2016 [11]
Inpatient procedures	-	Swiss federal regulation exists from 2019 [12]
Caesarean section	-	Swiss national guideline since 2015 [13]
AMI: aspirin	[14,15]	
AMI: statin	[14,15]	
AMI: beta-blocker	[14,15]	
AMI: ACE/ARB	[14,15]	
AMI: P2Y12 inhibitors	[14,15]	
PPI with NSAID	[16]	
PAD: statin	[17]	
Afib: anticoagulation	[18]	
GCC with new DMARD	[19]	

DM – diabetes mellitus, HbA1c – Glycated haemoglobin, LDL – low density lipid, TSH – thyroid-stimulating hormone, POCR – preoperative chest radiography, AMI – acute myocardial infarction, ACE/ARB – angiotensin-converting enzyme inhibitors or angiotensin II receptor blockers, PPI – proton pump inhibitors, NSAID – nonsteroidal anti-inflammatory drugs, PAD – peripheral artery disease, Afib – atrial fibrillation, GCC – glucocorticosteroid drugs, DMARD – disease-modifying antirheumatic drug.

1. Lansdorp-Vogelaar I, Karsa L. European guidelines for quality assurance in colorectal cancer screening and diagnosis. First Edition – Introduction. Endoscopy. 2012 Sep 25;44(S 03):SE15–30.
2. Screening for Breast Cancer: U.S. Preventive Services Task Force Recommendation Statement. Ann Intern Med. 2009 Nov 17;151(10):716.
3. Heidenreich A, Bellmunt J, Bolla M, Joniau S, Mason M, Matveev V, et al. EAU Guidelines on Prostate Cancer. Part 1: Screening, Diagnosis, and Treatment of Clinically Localised Disease. Eur Urol. 2011 Jan;59(1):61–71.

For peer review only

4. Kanis JA, McCloskey E V., Johansson H, Cooper C, Rizzoli R, Reginster J-Y. European guidance for the diagnosis and management of osteoporosis in postmenopausal women. Osteoporos Int. 2013 Jan 19;24(1):23–57.
5. Rydén L, Grant PJ, Anker SD, Berne C, Cosentino F, Danchin N, et al. ESC Guidelines on diabetes, pre-diabetes, and cardiovascular diseases developed in collaboration with the EASD. Eur Heart J. 2013 Oct 14;34(39):3035–87.
6. Bahn RS, Burch HB, Cooper DS, Garber JR, Greenlee MC, Klein I, et al. Hyperthyroidism and Other Causes of Thyrotoxicosis: Management Guidelines of the American Thyroid Association and American Association of Clinical Endocrinologists. Thyroid. 2011 Jun;21(6):593–646.
7. Garber J, Cobin R, Gharib H, Hennessey J, Klein I, Mechanick J, et al. Clinical Practice Guidelines for Hypothyroidism in Adults: Cosponsored by the American Association of Clinical Endocrinologists and the American Thyroid Association. Endocr Pract. 2012 Nov;18(6):988–1028.
8. Choosing Wisely. American College of Surgeons. Admission pre-op chest x-rays. September 4, 2013. Available from: https://www.choosingwisely.org/clinician-lists/american-college-surgeons-admission-or-preop-chest-x-ray-on-ambulatory-patients/ Accessed on May 11, 2020
9. Swiss Federal Office of Public Health. Recommendations for Influenza Vaccination (Empfehlungen zur Grippeimpfung). 2011.
10. Choosing Wisely. American Geriatrics Society. Benzodiazepines sedative hypnotics for insomnia in older adults. February 21, 2013. Available from: https://www.choosingwisely.org/clinician-lists/american-geriatrics-society-benzodiazepines-sedative-hypnotics-for-insomnia-in-older-adults/ Accessed on May 11, 2020
11. Swiss Society of General Internal Medicine. Smarter Medicine. Top-5 List for Ambulatory Care. 2016. Available from: https://www.smartermedicine.ch/de/top-5-listen/ambulante-allgemeine-innere-medizin.html Accessed on May 11, 2020
12. Swiss Federal Office of Public Health. "Outpatient instead of Inpatient" [Änderung der Krankenpflege-Leistungsverordnung (KLV) betreffend «Ambulant vor Stationär»]. Available from: https://www.bag.admin.ch/bag/de/home/versicherungen/krankenversicherung/krankenversicherung-revisionsprojekte/konsultation-ambulant-vor-stationaer.html Accessed on May 11, 2020
13. Hoesli I, Alma-Stucki S El, Drack G, Girard T, Irion O, Schulzke S, et al. Guideline Sectio Caesarea. 2015;1–20. Available from: https://www.sggg.ch/fachthemen/guidelines/ Accessed on May 11, 2020
14. Hamm CW, Bassand J-P, Agewall S, Bax J, Boersma E, Bueno H, et al. ESC Guidelines for the management of acute coronary syndromes in patients presenting without persistent ST-segment elevation: The Task Force for the management of acute coronary syndromes (ACS) in patients presenting without persistent ST-segment elevatio. Eur Heart J. 2011 Dec 1;32(23):2999–3054.
15. Steg PG, James SK, Atar D, Badano LP, Lundqvist CB, Borger MA, et al. ESC Guidelines for the management of acute myocardial infarction in patients presenting with ST-segment elevation. Eur Heart J. 2012 Oct 1;33(20):2569–619.
16. Recommendations for use of selective and nonselective nonsteroidal antiinflammatory drugs: An American College of Rheumatology white paper. Arthritis Rheum. 2008 Aug 15;59(8):1058–73.

For peer review only

17. Tendera M, Aboyans V, Bartelink M-L, Baumgartner I, Clement D, Collet J-P, et al. ESC Guidelines on the diagnosis and treatment of peripheral artery diseases: Document covering atherosclerotic disease of extracranial carotid and vertebral, mesenteric, renal, upper and lower extremity arteries * The Task Force on the Diagnosis and Treat. Eur Heart J. 2011 Nov 2;32(22):2851–906.

18. Camm AJ, Lip GYH, De Caterina R, Savelieva I, Atar D, Hohnloser SH, et al. 2012 focused update of the ESC Guidelines for the management of atrial fibrillation. Eur Heart J. 2012 Nov 1;33(21):2719–47.

19. Smolen JS, Landewé R, Breedveld FC, Buch M, Burmester G, Dougados M, et al. EULAR recommendations for the management of rheumatoid arthritis with synthetic and biological disease-modifying antirheumatic drugs: 2013 update. Ann Rheum Dis. 2014 Mar;73(3):492–509.

For peer review only

Additional file 5 Geographic variation of the health care services grouped by strength and direction of recommendations, and service category

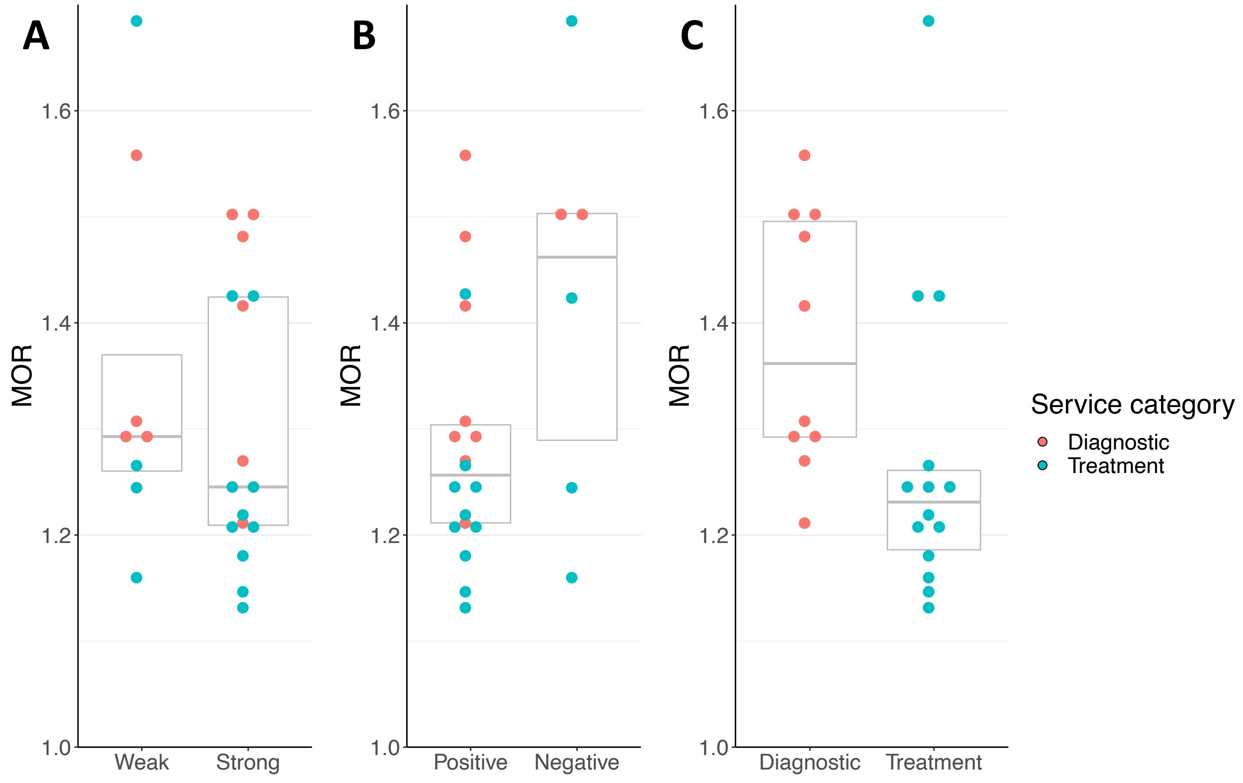

A Weak and strong recommendations; B Positive and negative recommendations; C Diagnostic and treatment services. MOR – median odds ratio. Boxplots depict the interquartile range of values (upper and lower hinges), and the median value.

Based on Welch's t-test, the difference in mean variances [95CI%] of diagnostic and treatment services was 0.04 [-0.01, 0.11], and the difference in mean MOR was 0.11 [-0.01, 0.23].

For peer review only - http://bmjopen.bmj.com/site/about/guidelines.xhtml

STROBE 2007 (v4) Statement—Checklist of items that should be included in reports of *cross-sectional studies*

Section/Topic	Item #	Recommendation	Reported on page #
**Title and abstract**	1	(*a*) Indicate the study's design with a commonly used term in the title or the abstract	1,2
		(*b*) Provide in the abstract an informative and balanced summary of what was done and what was found	2
**Introduction**			
Background/rationale	2	Explain the scientific background and rationale for the investigation being reported	4
Objectives	3	State specific objectives, including any prespecified hypotheses	4,5
**Methods**			
Study design	4	Present key elements of study design early in the paper	2,
Setting	5	Describe the setting, locations, and relevant dates, including periods of recruitment, exposure, follow-up, and data collection	5-7
Participants	6	(*a*) Give the eligibility criteria, and the sources and methods of selection of participants	7, Supplementary file 1
Variables	7	Clearly define all outcomes, exposures, predictors, potential confounders, and effect modifiers. Give diagnostic criteria, if applicable	7, Supplementary file 1
Data sources/ measurement	8*	For each variable of interest, give sources of data and details of methods of assessment (measurement). Describe comparability of assessment methods if there is more than one group	6, Supplementary files 1-4
Bias	9	Describe any efforts to address potential sources of bias	6,7
Study size	10	Explain how the study size was arrived at	5
Quantitative variables	11	Explain how quantitative variables were handled in the analyses. If applicable, describe which groupings were chosen and why	7
Statistical methods	12	(*a*) Describe all statistical methods, including those used to control for confounding	7
		(*b*) Describe any methods used to examine subgroups and interactions	7
		(*c*) Explain how missing data were addressed	NA
		(*d*) If applicable, describe analytical methods taking account of sampling strategy	NA
		(*e*) Describe any sensitivity analyses	NA
**Results**			

For peer review only

Participants	13*	(a) Report numbers of individuals at each stage of study—eg numbers potentially eligible, examined for eligibility, confirmed eligible, included in the study, completing follow-up, and analysed	8,9
		(b) Give reasons for non-participation at each stage	NA
		(c) Consider use of a flow diagram	NA
Descriptive data	14*	(a) Give characteristics of study participants (eg demographic, clinical, social) and information on exposures and potential confounders	9
		(b) Indicate number of participants with missing data for each variable of interest	NA
Outcome data	15*	Report numbers of outcome events or summary measures	9
Main results	16	(*a*) Give unadjusted estimates and, if applicable, confounder-adjusted estimates and their precision (eg, 95% confidence interval). Make clear which confounders were adjusted for and why they were included	9, Supplementary file 1
		(*b*) Report category boundaries when continuous variables were categorized	NA
		(*c*) If relevant, consider translating estimates of relative risk into absolute risk for a meaningful time period	NA
Other analyses	17	Report other analyses done—eg analyses of subgroups and interactions, and sensitivity analyses	NA
**Discussion**			
Key results	18	Summarise key results with reference to study objectives	11
Limitations	19	Discuss limitations of the study, taking into account sources of potential bias or imprecision. Discuss both direction and magnitude of any potential bias	13,14
Interpretation	20	Give a cautious overall interpretation of results considering objectives, limitations, multiplicity of analyses, results from similar studies, and other relevant evidence	14,15
Generalisability	21	Discuss the generalisability (external validity) of the study results	14
**Other information**			
Funding	22	Give the source of funding and the role of the funders for the present study and, if applicable, for the original study on which the present article is based	15

*Give information separately for cases and controls in case-control studies and, if applicable, for exposed and unexposed groups in cohort and cross-sectional studies.

Note: An Explanation and Elaboration article discusses each checklist item and gives methodological background and published examples of transparent reporting. The STROBE checklist is best used in conjunction with this article (freely available on the Web sites of PLoS Medicine at http://www.plosmedicine.org/, Annals of Internal Medicine at http://www.annals.org/, and Epidemiology at http://www.epidem.com/). Information on the STROBE Initiative is available at www.strobe-statement.org.

For peer review only
